# Artificial intelligence support for diagnosis of neurodevelopmental disorders during childhood: an umbrella review

**DOI:** 10.3389/fpsyt.2026.1697185

**Published:** 2026-03-18

**Authors:** Alejandro Alberca-González, Eduardo Fernández-Jiménez

**Affiliations:** 1 Faculty of Law, Education and Humanities, Universidad Europea de Madrid, Madrid, Spain; 2 Department of Child and Adolescent Psychiatry, Clinical Psychology and Mental Health, La Paz University Hospital, Madrid, Spain; 3 Hospital La Paz Institute for Health Research (IdiPAZ), Madrid, Spain

**Keywords:** artificial intelligence, childhood, diagnosis, neurodevelopmental disorders, umbrella review

## Abstract

**Introduction:**

The growing demand for earlier diagnosis of neurodevelopmental disorders has boosted critical assessment of artificial intelligence (AI) as a complementary tool for clinical decision-making.

**Methods:**

This umbrella review aimed to synthesize the available evidence from systematic reviews and meta-analyses on the use of AI to diagnose during childhood any neurodevelopmental disorder [autism spectrum disorder (ASD), attention-deficit/hyperactivity disorder (ADHD), intellectual disability, communication disorders, developmental coordination disorder, and specific learning disorders]. A systematic search was conducted on the Web of Science, PsycINFO, and PubMed, covering studies published from January 2015 to August 2025 and available in any language.

**Results:**

Of the 148 records identified, 64 studies were included based on the predefined inclusion and exclusion criteria. ASD (*n* = 31) and ADHD (*n* = 14) were the most frequently examined conditions in which AI was applied for diagnostic purposes. To a lesser extent, it was applied to specific learning disorders (*n* = 5) and other developmental disorders (intellectual disability and communication disorders, jointly addressed along with other diagnoses, *n* = 9). The most employed AI models were machine learning (support vector machines and artificial neural networks) and particularly deep learning (such as convolutional neural networks). These models were applied to diverse data modalities, such as neuroimaging (*n* = 59 studies), electrophysiological (*n* = 19), clinical/sociodemographic (*n* = 15), and motion/sensor-based data (*n* = 11). Overall, these AI models achieved diagnostic accuracy levels ranging from 66% (based on head/facial/eye movements) to 99% (based on neuroimaging, voice, motion, and sensors). However, the methodological quality of most studies was rated as critically low according to the AMSTAR-2 criteria (80%), while only 5% of studies achieved high quality levels (focused on ASD and ADHD).

**Conclusion:**

AI shows promising potential for supporting biomarker identification and diagnosis of neurodevelopmental disorders. However, future clinical implementation still requires methodologically rigorous research addressing current limitations: insufficient external validation, lack of standardization in data collection and model development, as well as reporting inconsistencies.

**Systematic Review Registration:**

https://www.crd.york.ac.uk/PROSPERO/view/CRD420251110825, identifier CRD420251110825.

## 1 Introduction

In clinical and healthcare settings, diagnosis of neurodevelopmental disorders at an early age poses considerable clinical challenges (1). In particular, some of the challenges faced by health professionals when coping with diagnosis and differential diagnosis, based on reference manuals such as the DSM-5-TR (2) or ICD-11 (3), fall into the following four major categories. On the one hand, professionals are faced with a relevant overlap of symptoms and disorders, such as attention-deficit/hyperactivity disorder (ADHD), autism spectrum disorder (ASD), global developmental delay, and communication disorders, which can share clinical manifestations, an aspect that is especially common in preschool stages, which could make it difficult to distinguish between them (4–6). Related to this, health professionals also have to deal with high comorbidity between disorders, that is, potential disorder co-presentation that complicates the precise identification of each clinical condition. Another aspect to consider when diagnosing neurodevelopmental conditions is the variability in the symptomatic evolution of the disorder (4, 5), which implies that some symptoms may be hidden or even disappear and emerge over time, thus requiring periodic longitudinal assessments. Finally, external factors such as access to services, parental education level, and socioeconomic environment could be key in the detection and modulation of neurodevelopmental disorders, which could generate inequalities in diagnosis, management, and prognosis depending on these contextual factors (7, 8). Therefore, the diagnosis and differential diagnosis of these neurodevelopmental conditions pose an important challenge for health professionals, influenced, among others, by symptomatic heterogeneity, symptom overlap, and external factors (such as social and contextual factors).

In this context, artificial intelligence (AI) has emerged as a promising tool that could improve diagnostic accuracy and facilitate screening in clinical and research settings (9). For example, Google’s recent development of a new AI model called *MedGemma* (10), created and trained specifically to understand and reason about medical texts and images, could facilitate aspects such as diagnosis, reporting, interpretation of diagnostic tests, and medical record analysis. The release of *MedGemma* under the OpenSource license model may facilitate healthcare centers and researchers adapting and fine-tuning this AI tool to meet specific requirements. This adaptability includes addressing aspects such as privacy in environments with sensitive clinical data, which could potentially lead to a significant rise in the utilization of these increasingly accessible tools.

However, despite the optimism surrounding these emerging tools, owing to the diversity of AI models used and their validity issues related to them, an updated systematic analysis is warranted to review the knowledge accumulated to date, addressing both the practical usefulness and current limitations of AI models applied in this field.

Given the above, the present umbrella review is the first to exhaustively synthesize systematic reviews and meta-analyses regarding the utilization of AI models in the diagnosis of all and specific neurodevelopmental disorders. To do so, this umbrella review focuses specifically on the pediatric population up to 12 years of age. This age range was selected to prioritize the critical developmental windows where diagnostic support is most needed. Epidemiological evidence indicates that the onset of neurodevelopmental disorders peaks at approximately 5–6 years, with 61.5% of cases emerging before the age of 14 (11). By restricting the scope to 12 years, this review encompasses two pivotal stages: the early identification of developmental delays (e.g., ASD, cerebral palsy) during the first 3–4 years (12, 13) and the diagnosis of school-age disorders (e.g., ADHD, specific learning disorders), which are most frequently identified between 6 and 9 years (8, 14). Therefore, focusing on this period is clinically essential, as guidelines emphasize that screening and intervention yield the best outcomes when initiated during these stages of maximal neuroplasticity (15, 16).

Given these diagnostic complexities and the rapid evolution of AI technologies, a comprehensive synthesis examining AI applications across all neurodevelopmental disorders is warranted to support clinical decisions.

## 2 Materials and methods

### 2.1 Design and eligibility criteria

An umbrella review was conducted following the Preferred Reporting Items for Overviews of Reviews (PRIOR) (17), the Preferred Reporting Items for Systematic Reviews and Meta-analyses (PRISMA) (18), and recommendations from the Joanna Briggs Institute’s (JBI) methodology working group (19). According to the PICO components (20), in this umbrella review were set the following:

-Population: children with neurodevelopmental disorders.
-Intervention: any AI model for diagnosis, diagnostic accuracy, screening, detection, risk assessment, and/or predictive purposes.
-Comparator: traditional methods against AI-based models.
-Outcome: any data modality (genetic/molecular, neuroimaging, electrophysiological, neuropsychological, sociodemographic, clinical, behavioral data, and others).

We screened studies from the initial literature pool according to the following inclusion and exclusion criteria for eligibility:

#### 2.1.1 Inclusion criteria

-Clinical condition: any neurodevelopmental disorder.
-Age: children (under 12 years of age).
-Methodology and techniques: any AI models.
-Article/document type: systematic reviews and/or meta-analysis.

#### 2.1.2 Exclusion criteria

-Clinical condition: neurological conditions or mental disorders other than neurodevelopmental disorders.
-Age: older than 12 years (adolescent and adult populations).
-Methodology and techniques: not AI-based models.
-Article/document type: study types other than systematic reviews and/or meta-analysis, such as primary studies, narrative literature, critical review, theoretical review, etc.

### 2.2 Search strategy and data sources

First, PROSPERO was checked for ongoing or already published systematic reviews on the subject, and out of the 53 results identified, no study covered the same aim or the same populations as this umbrella review. Consequently, this umbrella review was registered in PROSPERO (24 Jul 2025), where the entire search and selection process was documented (PROSPERO ID CRD420251110825).

A comprehensive bibliographic search was conducted in PubMed/MEDLINE, PsycINFO, and Web of Science (WoS). This specific combination was selected to ensure optimal coverage of the intersection between pediatric health, behavioral sciences, and computational applications, following the methodological recommendations for maximizing recall in systematic reviews (21). PubMed was included as the core biomedical resource to capture high-quality clinical and observational studies in pediatric populations (22, 23). PsycINFO was used to address the behavioral, cognitive, and mental health dimensions that are often underrepresented in purely biomedical indices (24, 25). Finally, the Web of Science was searched to broaden the scope of interdisciplinary fields, including medical informatics, and to leverage citation tracking, which significantly increases the retrieval of relevant studies compared to using MEDLINE alone (26, 27). The search aimed to obtain studies that addressed both AI (according to diverse models) and diagnosis/prediction of neurodevelopmental disorders globally, combining the following search string with controlled vocabulary from MeSH:

*(“Artificial Intelligence” OR “machine learning” OR “deep learning” OR “neural networks” OR “AI”) AND (“Neurodevelopmental Disorders” OR “autism* sp*ectrum disorder” OR “ADHD” OR “intellectual disability” OR “communication disorders” OR “language disorders” OR “developmental coordination disorder” OR “specific learning disorder”) AND (“Diagnosis” OR “Prediction” OR “predictive modeling” OR “early detection” OR “diagnostic accuracy” OR “risk assessment” OR “screening”).*

#### 2.2.1 Filters applied within the search strategy

-Article/document type, according to each database’s possibilities: *Web of Science* (Review Article); PubMed (Meta-Analysis and Systematic Review); and PsycINFO (not applicable after 0 results).
-Publication date/years: last 10 years, between 2015 and 2025.
-No language restrictions.

The last search was conducted in August 2025. Duplicates were then removed, and titles and abstracts were screened, followed by a full-text review. The selection and screening process were performed using the Covidence online application with two independent reviewers (AAG & EFJ), and discrepancies were resolved by consensus.

### 2.3 Data extraction and synthesis

After the final selection of studies, data extraction was independently performed by both authors, with main data extraction responsibility conducted by one author (AAG) and subsequently completed, revised, and validated by the other one (EFJ) until achieving consensus. Although the Covidence tool allows this to be done, owing to the flexibility limitations of this platform, we opted to record the data on an external spreadsheet, which was used for the remaining analyses.

For each study included, information was collected on countries of publication, the disorder addressed, the type of AI model applied and data source, measures of diagnostic efficacy, limitations highlighted, and main conclusions of each study (see **Supplementary Table 1**).

#### 2.3.1 Handling of overlapping primary studies

Given that umbrella reviews synthesize systematic reviews that may include overlapping primary studies, we adopted the following approach to minimize any bias. First, we documented the degree of overlap by identifying the primary studies cited across multiple included reviews. Second, when synthesizing quantitative findings, we prioritized meta-analyses with the largest sample sizes and the most recent publication dates to reduce redundancy. Third, for qualitative synthesis, we focused on converging conclusions across reviews rather than aggregating individual effect sizes, thereby minimizing the influence of multiply-counted studies.

### 2.4 Assessment of methodological quality and bias risk

The studies included in this umbrella review were critically assessed for their methodological quality. Double appraisal was conducted independently by the two authors of this work, and any discrepancies were resolved by consensus. This critical appraisal was performed using the “*A MeaSurement Tool to Assess Systematic Reviews*,” 2nd edition (AMSTAR-2) (28). This updated version contains 16 items, where 7 are critical (Items 2, 4, 7, 9, 11, 13, and 15). To achieve a rating of *high* quality, studies must have no critical flaws and less than two non-critical weaknesses; a rating of *moderate* quality requires no critical weakness; *low* quality requires only one critical weakness; and *critically low* quality implies more than one critical weakness.

For this umbrella review, AMSTAR-2 was adjusted as follows: Item (1) regarding PICO components, letter *I* will refer to diagnostic/predictive tools instead of interventions; Item (3), it is non-critical and was not considered because it refers to different study designs (randomized and/or non-randomized trials), which is not applicable in this umbrella review; Item (7) was codified as “yes” if the reasons for excluded studies were clarified in the text or in the PRISMA flowchart.

## 3 Results

### 3.1 Study selection

**Figure 1:**
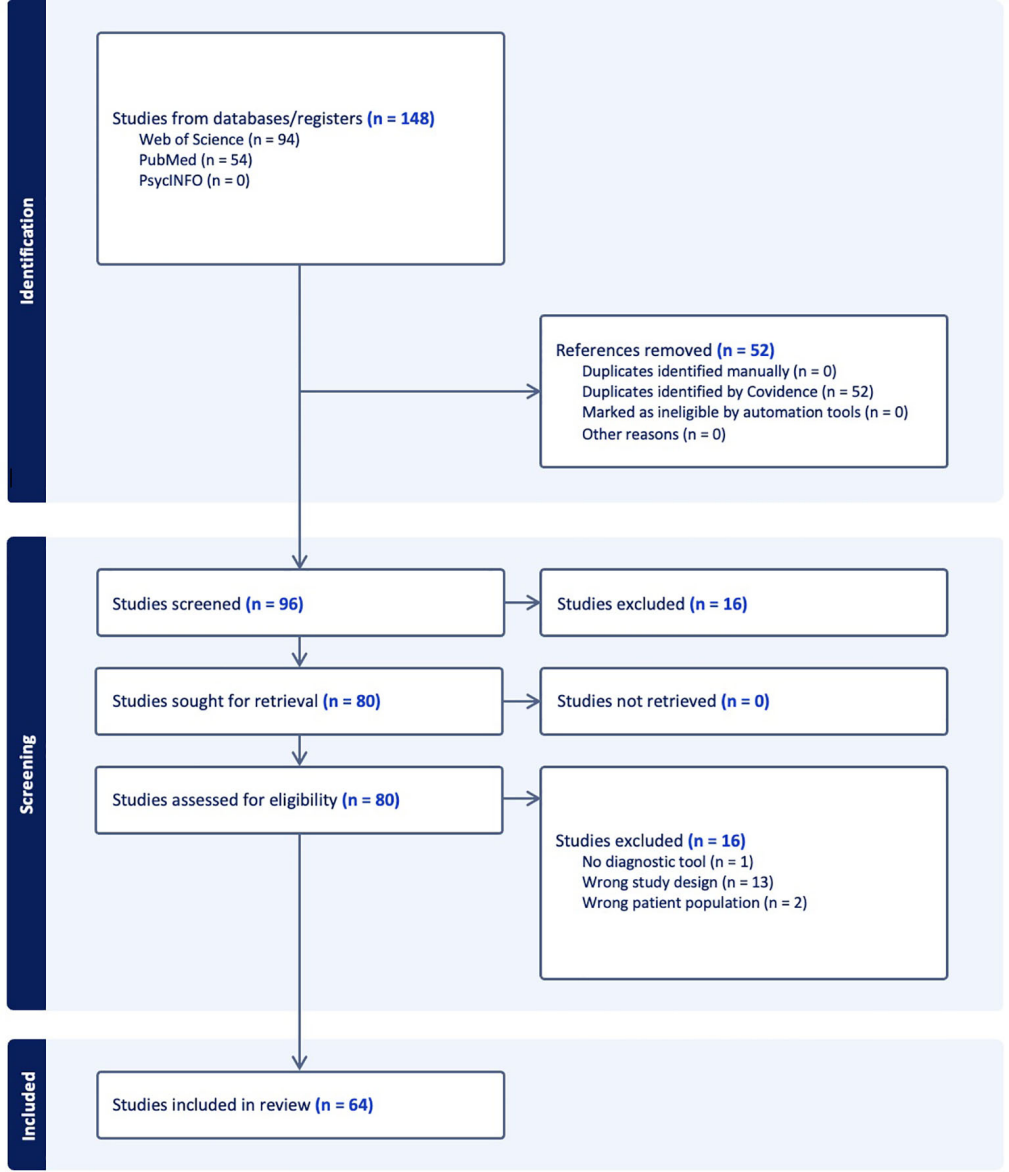
PRISMA study selection flowchart.

The flow of the selection process is detailed in the PRISMA flowchart in **Figure 1**. A total of 148 studies were identified, of which 64 systematic reviews and/or meta-analyses were included after applying the inclusion and exclusion criteria. Sixteen studies were excluded for the following reasons: one study did not address diagnostic methods or tools, thirteen studies employed an incorrect study design (not systematic reviews or meta-analyses), and two studies included an inappropriate patient population (see **Supplementary Table 2**). While the majority of the articles were published in English, two articles were published in Spanish, and another in Chinese. Clinical conditions other than neurodevelopmental disorders were not examined for this umbrella review when this data was also present in the studies included (e.g., neurological diseases, neurogenetic conditions, and other mental disorders).

### 3.2 Data extraction and summary of results

#### 3.2.1 Time evolution of publications

**Figure 2:**
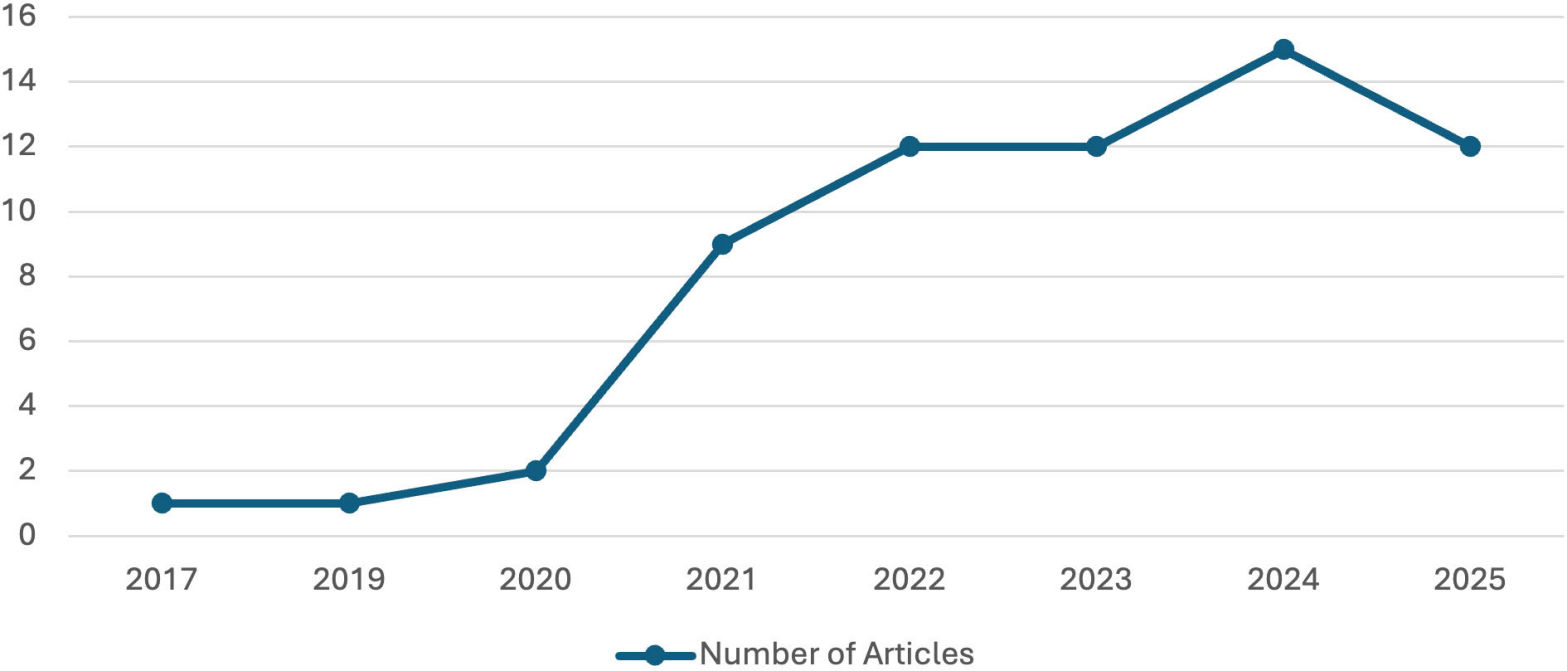
Time evolution of studies included according to publication year.

The number of systematic reviews and meta-analyses on the use of AI in the diagnosis of neurodevelopmental disorders has increased significantly since 2017, as can be seen in **Figure 2**. A significant increase in the number of published studies is observed from 2021 until it reaches its peak in 2024 (see **Table 1**).

**Table 1:** Time evolution of studies included according to publication year.

Publication year	Number of studies	Studies included
2017	1	Fusaroli et al. (29)
2019	1	Valliani et al. (30)
2020	2	Geng et al. (31); Rahman et al. (32)
2021	9	Patel et al. (33); Cavus et al. (34)Miranda et al. (35)Pereira-Sánchez & Castellanos (36)Quaak et al. (37)Senior et al. (38)Silva et al. (39)Song et al. (40)Zhang et al. (41)
2022	11	Alam et al. (42)Alqaysi et al. (43)Alves et al. (44)Dwyer & Koutsouleris (45)Francese & Yang (46)Kang et al. (47)Kohli et al. (48); Mengi & Malhotra (49); Moridian et al. (50)Santana et al. (51)Welch et al. (52)
2023	12	Alharthi & Alzahrani (53)Chen et al. (54)Cruz et al. (55)Das et al. (56)Hu et al. (57)Iyortsuun et al. (58)Joudar et al. (59)Khare et al. (60)Parlett-Pelleriti et al. (61)Ribas et al. (62); Salgado et al. (63); Zhang-James et al. (64)
2024	15	Ding et al. (9)Banos et al. (65)Fatima & Masood (66)Huda et al. (67); Lee et al. (6); Li et al. (68)Quintero et al. (69)Rajagopalan & Tammimies (70)Rogers et al. (71)Simeoli et al. (72)Swinckels et al. (73)Tian et al. (74)Toki (75)Uddin et al. (76)Wen et al. (77)
2025	12	Khan & Shang (1)Berchio et al. (78)Bouchouras & Kotis (79)Cerasuolo et al. (80)Ganggayah et al. (81)K.B. & P.M. (82)Rezaee (83)Sohn et al. (84); Solek et al. (85); Taneera & Alhajj (86)Vimbi et al. (87)Zaheer & Akhtar (88)

This trend reflects the growing interest in how the use of AI tools could contribute to the diagnosis of neurodevelopmental diseases.

#### 3.2.2 Geographical distribution of publications

**Figure 3:**
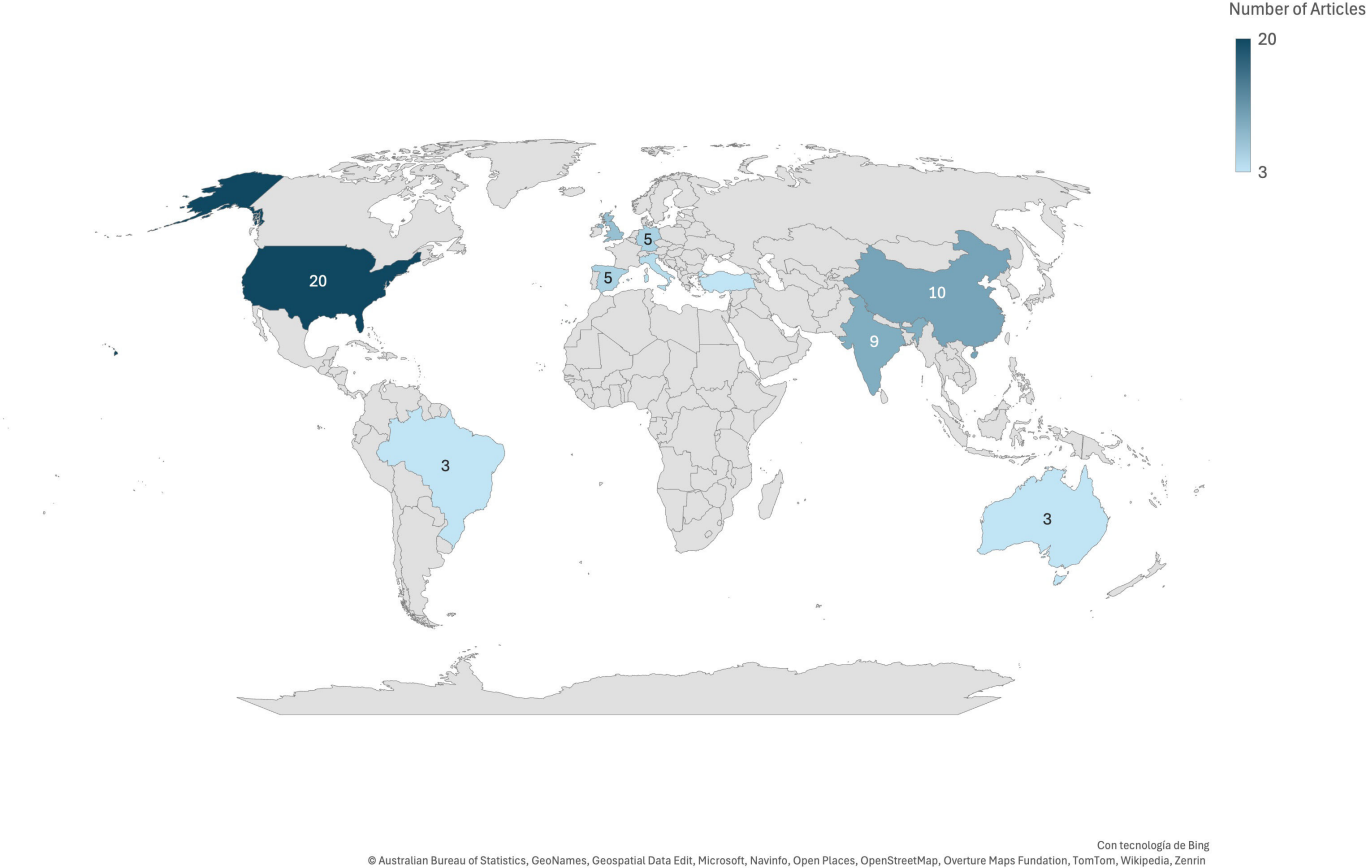
Map of geographical distribution of publications.

As can be seen in **Figure 3**, most of the included studies came from countries such as the United States, China, the United Kingdom, India, and South Korea, evidencing the concentration of research in regions with more technological resources and development in AI (1, 67, 81). However, it also shows the growing participation of other European countries, Latin America, and other regions, although in smaller proportions, reflecting that interest is not only growing but also increasingly globalized. **Table 2** presents detailed data for this outcome.

**Table 2:** Geographical distribution of studies included.

Country	Number of studies	Studies included
United States	20	Dwyer & Koutsouleris (45)Cruz et al. (55)Fatima & Masood (66)Bouchouras & Kotis (79); Francese & Yang (46); Geng et al. (31); Miranda et al. (35)Pereira-Sanchez & Castellanos (36)Senior et al. (38)Zhang et al. (41)Santana et al. (51)Welch et al. (52)Hu et al. (57)Joudar et al. (59)Parlett-Pelleriti et al. (61)Zhang-James et al. (64)Rogers et al. (71)Swinckels et al. (73)Uddin et al. (76)Wen et al. (77)
China	10	Ding et al. (9)Alharthi & Alzahrani (53); Geng et al. (31); Khan & Shang (1)Song et al. (40)Zhang et al. (41)Zhang-James et al. (64)Li et al. (68)Wen et al. (77)Vimbi et al. (87)
India	9	Alam et al. (42)Kohli et al. (48)Alharthi & Alzahrani (53)Khare et al. (60)Fatima & Masood (66)Huda et al. (67)K.B. & P.M. (82); Mengi & Malhotra (90); Vimbi et al. (87)
United Kingdom	7	Valliani et al. (30)Miranda et al. (35)Pereira-Sanchez & Castellanos (36)Senior et al. (38)Alqaysi et al. (43)Cruz et al. (55)Taneera & Alhajj (86)
Germany	5	Miranda et al. (35)Quaak et al. (37)Silva et al. (39)Dwyer & Koutsouleris (45)Santana et al. (51)
Spain	5	Pereira-Sanchez & Castellanos (36)Alves et al. (44)Banos et al. (65)Berchio et al. (78); Salgado et al. (63)
Italy	4	Miranda et al. (35)Ribas et al. (62)Cerasuolo et al. (80)de Barros et al. (89)
Turkey	3	Moridian et al. (50)Santana et al. (51)Parlett-Pelleriti et al. (61)
Brazil	3	Santana et al. (51)Ribas et al. (62)de Barros et al. (89)
Australia	3	Quintero-López (69); Santana et al. (51)Uddin et al. (76)
Others	15	Bangladesh: Vimbi et al. (87); Denmark: Fusaroli et al. (29); Greece: Khare et al. (60)Toki (75); Indonesia: Solek et al. (85); Iran: Li et al. (68)Rezaee (83); Malaysia: Rahman (32), Lee et al. (6); Oman: Vimbi et al. (87); Saudi Arabia: Alqaysi et al. (43)Alharthi & Alzahrani (53); South Korea: Kang (47)Sohn (84); Tunisia: Bouchouras & Kotis (79)

#### 3.2.3 Neurodevelopmental disorders addressed in the studies

**Figure 4:**
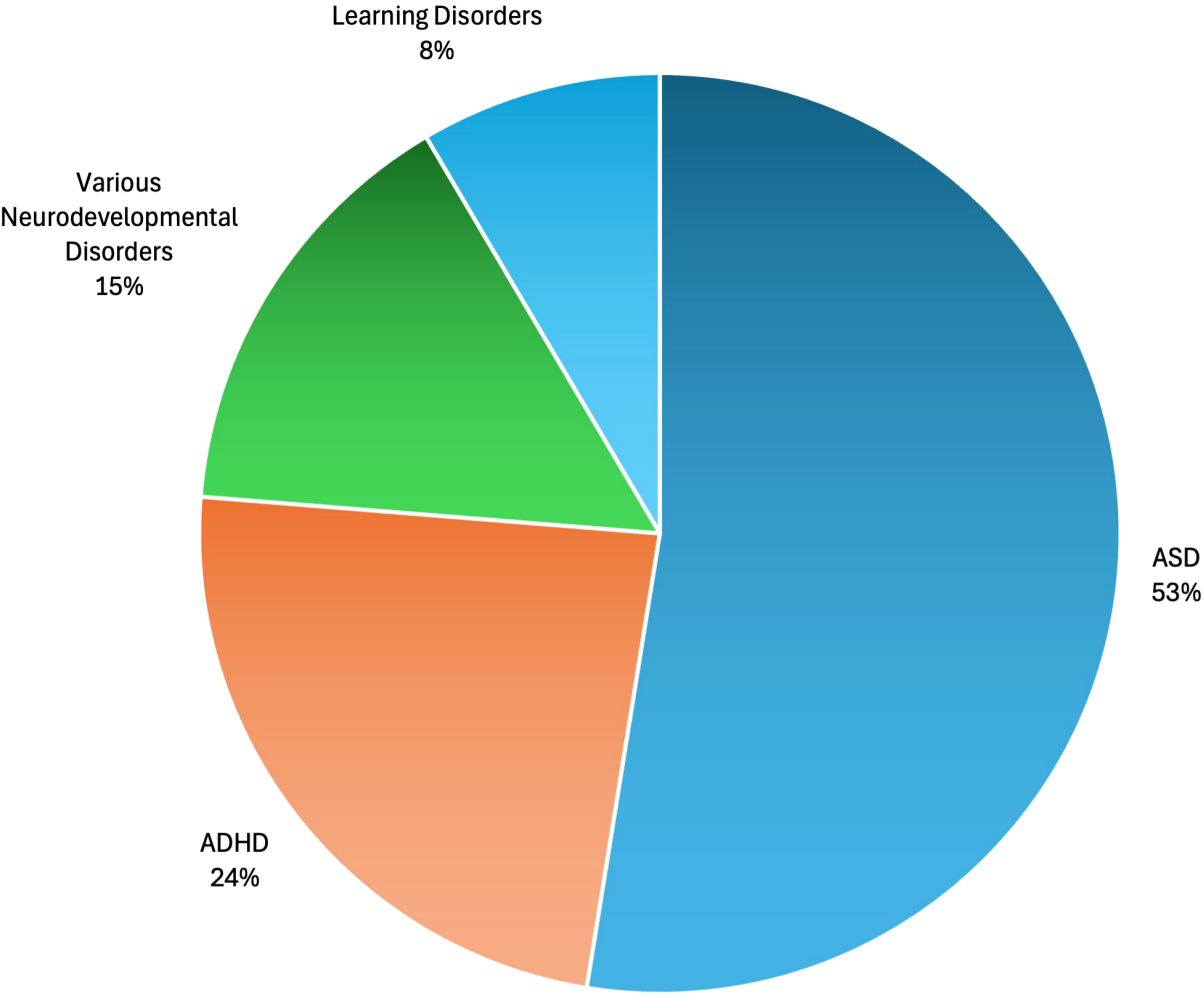
Percentage of studies included according to the neurodevelopmental disorders addressed.

Although neurodevelopmental disorders vary widely, after examining the studies included in detail (see **Figure 4**), 53% of the 64 selected systematic reviews and/or meta-analyses focused on ASD (*n* = 31), followed by ADHD (24%, *n* = 14). This was followed by various neurodevelopmental disorders (15%, *n* = 9). At 8%, studies on specific learning disorders were found (*n* = 5). Therefore, we can observe that, although there are disorders that are addressed predominantly (ASD and ADHD), there is also great variability in the disorders studied (see **Table 3**).

**Table 3:** Distribution of studies according to the neurodevelopmental disorder examined.

Disorder	Number of studies	Studies included
Autism Spectrum Disorder (ASD)	31	Ding et al. (9)Cavus et al. (34)Alqaysi et al. (43)Dwyer & Koutsouleris (45)Alharthi & Alzahrani (53)Chen et al. (54)Cruz et al. (55)Berchio et al. (78)Bouchouras & Kotis (79)Cerasuolo et al. (80)Ganggayah et al. (81)de Barros et al. (89); Geng et al. (31); Khan & Shang (1)Hu et al. (57)Joudar et al. (59)Khare et al. (60)Huda et al. (67); Mengi & Malhotra (90); Miranda et al. (91); Rahman et al. (32)Quaak et al. (37)Moridian et al. (50)Parlett-Pelleriti et al. (61); Solek et al. (85); Zhang et al. (41)Uddin et al. (76)Wen et al. (77)Sohn et al. (84)Taneera & Alhajj (86)Vimbi et al. (87)
Attention-Deficit/Hyperactivity Disorder (ADHD)	14	Alves et al. (44)Berchio et al. (78); Francese & Yang (46); Iyortsuun et al. (58); Lee et al. (6); Pereira-Sanchez & Castellanos (36); Quintero-López (69); Rahman et al. (32)Ribas et al. (62); Salgado et al. (63); Senior et al. (38)Zhang-James et al. (64)Tian et al. (74)Zaheer & Akhtar (88)
Various Neurodevelopmental Disorders jointly analyzed, including others (intellectual disability and communication disorders)	9	Alam et al. (42)Cruz et al. (55)Khare et al. (60)Fatima & Masood (66); López et al. (92); Valliani et al. (30)Silva et al. (39)Ribas et al. (62)Wei et al. (6)
Learning Disorders	5	Kang et al. (47)Cruz et al. (55)Khare et al. (60)Ribas et al. (62)Toki (75)

#### 3.2.4 AI models and types of data sources used

Various AI models have been used to interpret different types of data to improve diagnosis. Therefore, it was essential to analyze how this use has been done to better understand the functioning and functionality of AI in the studies analyzed, in which consistent patterns were revealed.

Classical machine learning models, such as SVM, Random Forest, k-NN, and decision trees, are predominantly applied to neuroimaging data (40, 41, 77), although they also have relevant applications in EEG/MEG analysis (68, 69), speech data (29, 59), motion captured by sensors (65, 72), and, to a lesser extent, clinical, sociodemographic, and facial information (33, 89).

Deep-learning models, particularly convolutional and recurrent neural networks, have shown a clear expansion in neuroimaging processing (9, 76), EEG signals (64, 78), and speech and motion analysis (39, 46, 71), allowing complex and multidimensional problems to be addressed.

However, the recent irruption of advanced architecture such as transformers and GNNs has been particularly remarkable in the processing of neuroimaging and text data (1, 53, 57), although they are still less frequent than traditional models. In addition, hybrid and data fusion approaches have emerged as promising alternatives for combining diverse sources of information (60, 69, 71, 77), suggesting a trend toward the development of more integrative and robust models.

These results reflect a clear association between model sophistication and the complexity and multimodality of the data employed, underscoring the need to move toward methodologies that allow for greater integration and interpretability in the clinical setting. This pattern suggests that AI innovation in neurodevelopment is not merely quantitative but a necessary response to the multidimensional nature of current biomarkers (see **Table 4**).

**Table 4:** Relationship between types of artificial intelligence models and data modalities in the diagnosis of neurodevelopmental disorders.

AI model	Neuroimaging (MRI, fMRI, DTI)	EEG/MEG	Voice/Acoustics	Motion/Sensors	EHR	Clinical/Sociodemographic	Facial imaging	Text	Eye tracking
SVM, Random Forest, k-NN, DT, Naive Bayes	24 studies	8 studies	3 studies	4 studies	3 studies	6 studies	2 studies	1 study	1 study
Deep Learning (CNN, RNN, LSTM, Autoencoders)	20 studies	8 studies	3 studies	5 studies	2 studies	5 studies	2 studies	2 studies	0
Transformers, GNN, Graph-based, BERT	5 studies	1 study Khan & Shang (1)	2 studies	0	1 study	0	0	1 study	0
Hybrid/Data Fusion	7 studies	2 studies Ribas et al. (62); Lee et al. (6)	2 studies	2 studies	3 studies	4 studies	2 studies	0	0
Unsupervised (clustering, PCA, UMAP, t-SNE)	3 studies	0	0	0	0	0	0	1 study	0

#### 3.2.5 Synthesis of diagnostic efficacy

**Figure 5:**
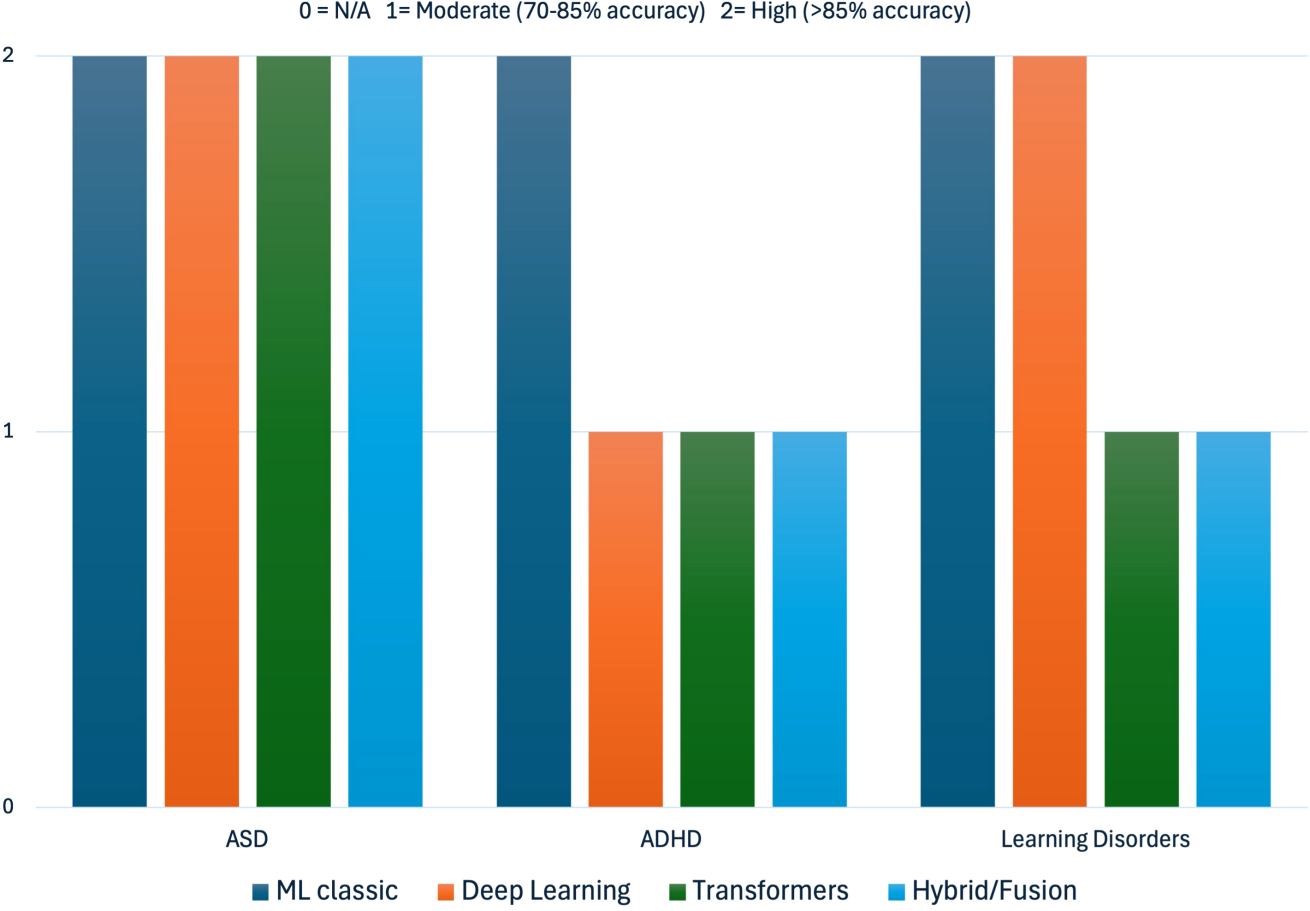
Comparative diagnostic efficacy of main AI models according to the neurodevelopmental disorder examined. Diagnostic efficacy ratings are derived from accuracy values reported in the included systematic reviews and meta-analyses, categorized as follows: High Efficacy (score = 2): Accuracy >90%, indicating strong discriminative performance in distinguishing individuals with the disorder from controls under study conditions. Moderate Efficacy (score = 1): Accuracy 70%–89%, indicating acceptable but variable performance that may require optimization for clinical use. Not Applicable/Insufficient Data (score = 0): No quantitative accuracy data reported, or studies were exclusively qualitative. These thresholds were established based on conventional benchmarks in diagnostic test evaluation literature and the distribution of accuracy values observed across included studies. Ratings reflect performance under research conditions and should not be directly extrapolated to clinical settings without external validation.

This umbrella review also examined which AI models were most effective in the diagnosis of neurodevelopmental disorders (see **Figure 5**).

In the case of **ASD**, 31 recent studies have confirmed a high diagnostic efficacy using diverse AI models. However, if we refer to specific models, we see that such efficacy is especially high in machine learning models (SVM, Random Forest, and k-NN), deep learning (CNN and autoencoders), and, to a lesser extent, transformer-type models. Accuracy usually exceeds 90% in neuroimaging and electrophysiological analysis (MRI, fMRI, EEG), achieving lower levels of accuracy (66%) before multimodal data of different domains (facial expressions, gaze tracking, eye tracking, and head pose estimation). However, methodological heterogeneity and lack of standardization limit the clinical generalization of these findings (1, 9, 40, 41, 77; among others).

For **ADHD**, 14 studies were included, with an average efficacy ranging from 80% to 92% in quantitative analyses, being somewhat lower or only “promising” in qualitative reviews. Classical models have shown the highest degree of effectiveness compared with those using deep learning, with no evidence of effectiveness regarding transformer-type models. The most frequent models include SVM, decision trees, random forest, and non-conventional methods, such as fuzzy logic or evolutionary algorithms, which are mainly applied to neuroimaging, EEG, and clinical or behavioral data. However, the variability in samples and methodological procedures makes comparison and clinical applicability difficult (58, 64, 69, 88).

For specific learning disorders, although there were few studies (*n* = 5), the results were consistent. In particular, moderate efficacy has been reported, with accuracy levels ranging between 70% and 88% for models based on SVM, k-NN, ANN, and deep learning, although clinical validation is still incipient (42, 62, 66) (see **Table 5**).

**Table 5:** Comparative cross-table summary of reviews: disorder, ai model type, data analyzed, and reported diagnostic efficacy.

Disorder	No. of reviews	Main AI models	Data type	Average efficacy	Critical comment	Studies included
Autism Spectrum Disorder (ASD)	31	Machine Learning (SVM, Random Forest, k-NN), Deep Learning (CNN, Autoencoders), Transformers	Neuroimaging (MRI, fMRI, EEG), clinical data, behavioral data, facial images	High in most cases (> 90% accuracy in MRI/EEG studies, but with high heterogeneity; in reviews without numerical data, ‘high efficacy’ is reported qualitatively)	AI shows high potential in ASD diagnosis, especially with neuroimaging and data fusion, though clinical validation and standardization remain limitations.	Ding et al. (9)Cavus et al. (34)Alqaysi et al. (43)Dwyer & Koutsouleris (45)Alharthi & Alzahrani (53)Chen et al. (54)Cruz et al. (55)Berchio et al. (78)Bouchouras & Kotis (79)Cerasuolo et al. (80)Ganggayah et al. (81)de Barros et al. (89); Geng et al. (31); Khan & Shang (1)Hu et al. (57)Joudar et al. (59)Khare et al. (60)Huda et al. (67); Mengi & Malhotra (49); Miranda et al. (91); Rahman (32)Quaak et al. (37)Moridian et al. (50)Parlett-Pelleriti et al. (61)Sohn (84); Solek et al. (85); Zhang et al. (41)Uddin et al. (76)Wen et al. (77)Taneera & Alhajj (86)Vimbi (87)
Attention-Deficit/Hyperactivity Disorder (ADHD)	14	Machine Learning (SVM, Decision Tree, Random Forest), non-conventional methods (fuzzy logic, evolutionary)	Neuroimaging, EEG, clinical scales, behavioral data	Medium-high (quantitative reviews: accuracy 80%–92%; qualitative: promising but no explicit numerical data)	AI supports ADHD diagnosis, but sample and method variability hampers generalizable application.	Alves et al. (44)Berchio et al. (78); Francese & Yang (46); Iyortsuun et al. (58); Lee et al. (6); Pereira-Sanchez & Castellanos (36); Quintero-López (69); Rahman (32)Ribas et al. (62); Salgado et al. (63); Senior et al. (38)Zhang-James et al. (64)Tian et al. (74)Zaheer & Akhtar (88)
Learning Disorders	5	Machine Learning (SVM, k-NN, ANN), Deep Learning	School data, clinical scales, neuroimaging, eye tracking	Moderate (reported accuracy 70%–88%; qualitative reviews suggest utility, but little clinical validation)	AI may be useful for learning disorders identification, but clinical use is still incipient.	Kang (47)Cruz et al. (55)Khare et al. (60)Ribas et al. (62)Toki (75)

#### 3.2.6 Limitations in AI models identified in the studies included

**Figure 6:**
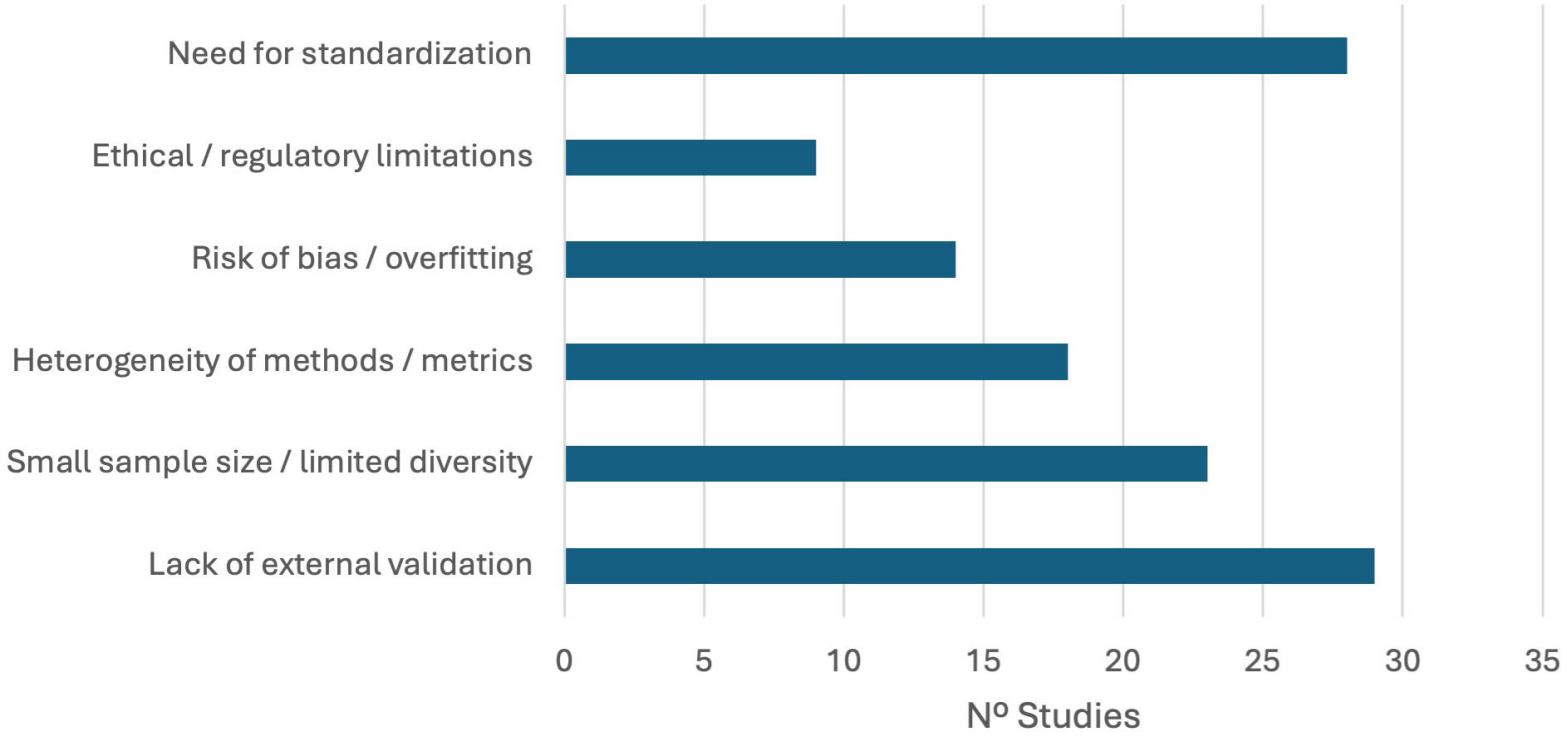
Frequency of main limitations reported in studies included. Bar lengths indicate the number of systematic reviews and meta-analyses that identified each limitation as a key barrier to the clinical implementation or generalizability of artificial intelligence models in the diagnosis of neurodevelopmental disorders.

Independent of the results obtained, it is important to reflect on the limitations encountered in the studies included to analyze the convenience of extrapolating and generalizing the data to other settings (see **Figure 6**). The critical analysis of the included systematic reviews and meta-analyses points out several recurrent limitations that hinder the clinical translation and robustness of AI models to be applied to the diagnosis of neurodevelopmental disorders. Lack of external validation was the most frequently identified barrier, noted in 32 articles (e.g., 34, 41, 45, 67, 90), followed closely by the need for standardization in data collection, model development, and reporting practice (31 articles; 57, 77, 92). Small sample sizes, low population diversity (26 studies; 50, 89), and heterogeneity of methods and metrics (21 studies; 9, 37) have also been widely identified, reflecting the persistent challenges in generalizability and comparability across studies.

In addition, 23% of the studies (*n* = 17) emphasized the risk of model bias and overfitting, which is often linked to insufficient validation protocols and reliance on single-center datasets (62, 66). In addition, ethical and regulatory issues were discussed in 12 studies, highlighting the importance of transparency, explainability, and regulatory oversight in future developments (35, 46, 73, 92).

Overall, these results indicate that despite technical advances and the seemingly promising diagnostic performance of AI models, the field still faces challenges related to methodological heterogeneity, the absence of large and diverse databases, the lack of external and prospective validation, and incipient ethical and regulatory issues (6, 38, 54) (**Table 6**).

**Table 6:** Limitations and challenges in diagnosis of neurodevelopmental disorders using AI models: thematic summary.

Theme	Number of studies	Studies included
Lack of external validation	32	Ding et al. (9)Cavus et al. (34)Alqaysi et al. (43)Dwyer & Koutsouleris (45)Chen et al. (54)Fatima & Masood (66)Bouchouras & Kotis (79); Francese & Yang (46); Kang et al. (47)Hu et al. (57)Huda et al. (67); Lee et al. (6); Mengi & Malhotra (90); Miranda et al. (35)Quaak et al. (37)Moridian et al. (50)Parlett-Pelleriti et al. (61); Quintero-López (69);Rahman et al. (32)Ribas et al. (62)Rajagopalan & Tammimies (70); Salgado et al. (63); Senior et al. (38)Silva et al. (39)Song et al. (40)Zhang et al. (41)Santana et al. (51)Swinckels et al. (73)Toki (75)Uddin et al. (76)Wen et al. (77)Taneera & Alhajj (86)Vimbi et al. (87)
Need for standardization	31	Ding et al. (9)Cavus et al. (34)Alqaysi et al. (43)Dwyer & Koutsouleris (45)Chen et al. (54)Fatima & Masood (66)Bouchouras & Kotis (79)de Barros et al. (89); Francese & Yang (46); Kang et al. (47)Hu et al. (57)Huda et al. (67); Lee et al. (6); Mengi & Malhotra (90); Miranda et al. (35)Quaak et al. (37)Moridian et al. (50)Parlett-Pelleriti et al. (61); Quintero-López (69);Rahman et al. (32)Ribas et al. (62); Salgado et al. (63); Silva et al. (39)Song et al. (40)Zhang et al. (41)Santana et al. (51)Toki (75)Uddin et al. (76)Wen et al. (77)Sohn et al. (84)Taneera & Alhajj (86)Vimbi et al. (87)
Small sample size/limited diversity	26	Ding et al. (9)Cavus et al. (34)Alqaysi et al. (43)Dwyer & Koutsouleris (45)Chen et al. (54)Fatima & Masood (66)de Barros et al. (89); Francese & Yang (46); Kang et al. (47)Hu et al. (57)Huda et al. (67); Mengi & Malhotra (90); Miranda et al. (35)Quaak et al. (37)Silva et al. (39)Song et al. (40)Zhang et al. (41)Moridian et al. (50)Santana et al. (51)Ribas et al. (62)Rajagopalan & Tammimies (70)Toki (75)Uddin et al. (76)Wen et al. (77)Taneera & Alhajj (86)Vimbi et al. (87)
Heterogeneity of methods/metrics	21	Ding et al. (9)Cavus et al. (34)Alqaysi et al. (43)Dwyer & Koutsouleris (45)Kang et al. (47)Hu et al. (57)Fatima & Masood (66)Huda et al. (67)de Barros et al. (89); Lee et al. (6); Mengi & Malhotra (90); Rahman et al. (32)Miranda et al. (35)Quaak et al. (37)Song et al. (40)Zhang et al. (41)Moridian et al. (50)Parlett-Pelleriti et al. (61)Ribas et al. (62)Taneera & Alhajj (86)Vimbi et al. (87)
Risk of bias/overfitting	17	Ding et al. (9)Alqaysi et al. (43)Dwyer & Koutsouleris (45)Chen et al. (54)Fatima & Masood (66); Francese & Yang (46); Kang et al. (47)Hu et al. (57); Mengi & Malhotra (90); Rahman et al. (32)Miranda et al. (35)Quaak et al. (37)Song et al. (40)Zhang et al. (41)Moridian et al. (50)Ribas et al. (62)Vimbi et al. (87)

#### 3.2.7 Assessment of methodological quality or bias risk in the studies included

**Figure 7:**
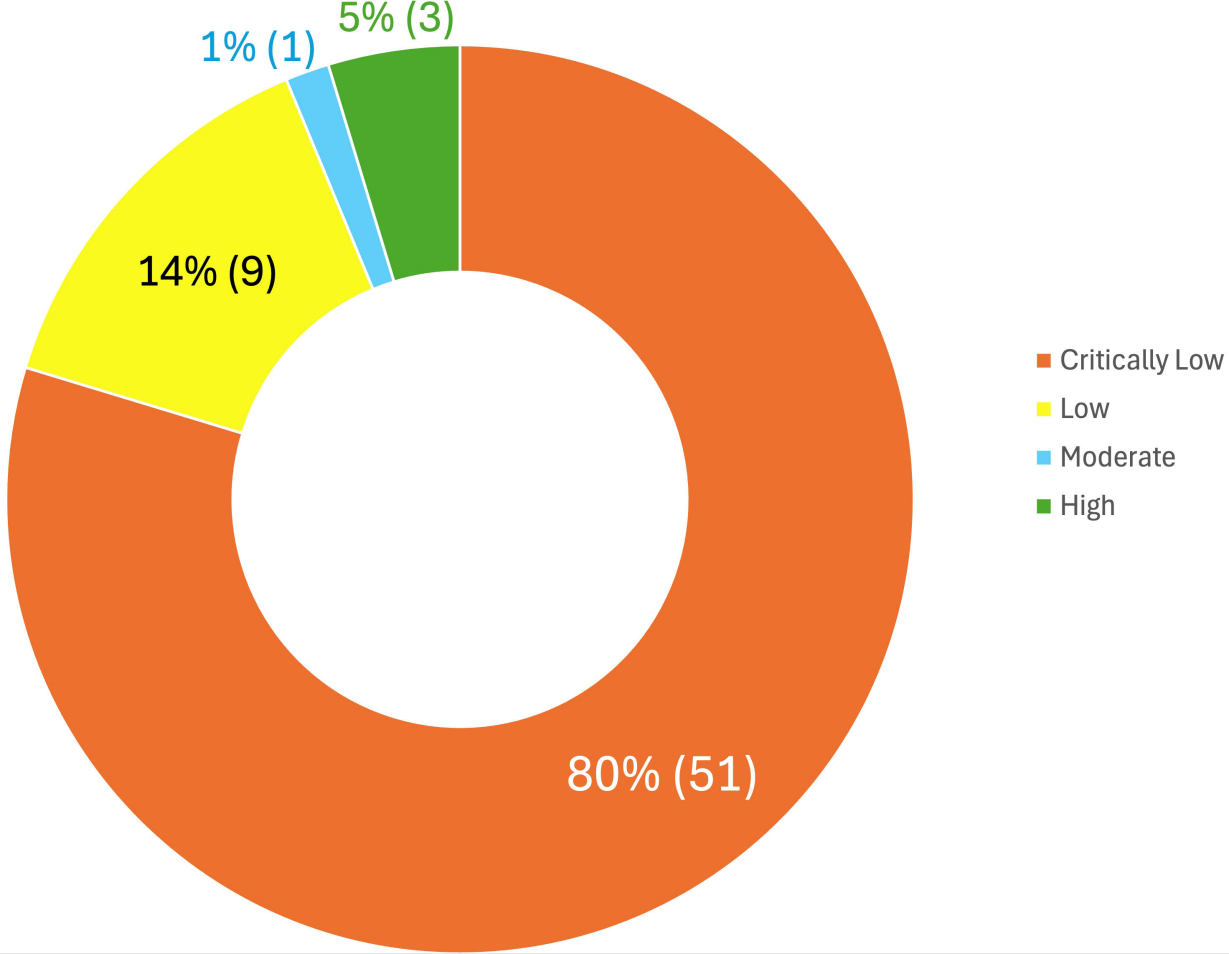
Methodological quality of studies included.

After critically appraising the studies included using the AMSTAR-2 tool, the majority were rated as critically low (80%) or low (14%) according to the instrument’s standardized criteria. It is important to note that AMSTAR-2 ratings reflect adherence to specific methodological reporting standards rather than an overall judgment of the scientific value of a particular study. In this sense, a ‘Critically Low’ rating indicates the presence of more than one critical weakness in domains such as protocol registration, comprehensive search strategies, or risk of bias assessment—common challenges in rapidly evolving fields like AI diagnostics where reporting standards are still being established (see **Table 7**, **Figure 7**). In contrast, only 1% achieved moderate levels, and 5% obtained high-quality levels. All these studies with better methodological quality analyzed ADHD and/or ASD, examining these conditions both exclusively in separate studies and jointly alongside other neurodevelopmental and mental disorders.

**Table 7:** Consensus on the methodological quality of the studies analyzed.

Study	Consensus
Alam et al. (42)	Critically Low
Alharthi & Alzahrani (53)	Critically Low
Alqaysi et al. (43)	Critically Low
Alves et al. (44)	Critically Low
Banos et al. (65)	Critically Low
Berchio et al. (78)	High
Bouchouras & Kotis (79)	Critically Low
Cavus et al. (34)	Critically Low
Cerasuolo et al. (80)	Critically Low
Chen et al. (54)	High
Cruz et al. (55)	Critically Low
Das et al. (56)	Low
De Barros et al. (89)	Critically Low
Ding et al. (9)	Low
Dwyer & Koutsouleris (45)	Critically Low
Fatima & Masood (66)	Critically Low
Francese & Yang (46)	Critically Low
Fusaroli et al. (29)	Critically Low
Ganggayah et al. (81)	Critically Low
Geng et al. (31)	Critically Low
Hu et al. (57)	Low
Huda et al. (67)	Critically Low
Iyortsuun et al. (58)	Critically Low
Joudar et al. (59)	Critically Low
K.B. & P.M. (82)	Critically Low
Kang et al. (47)	Low
Khan & Shang (1)	Critically Low
Khare et al. (60)	Critically Low
Kohli et al. (48)	Low
Li et al. (68)	Critically Low
Mengi & Malhotra (49)	Critically Low
Mengi & Malhotra (90)	Critically Low
Miranda et al. (35)	Critically Low
Moridian et al. (50)	Critically Low
Parlett-Pelleriti et al. (61)	Critically Low
Pereira-Sanchez & Castellanos (36)	Critically Low
Quaak et al. (37)	Critically Low
Quintero-López et al. (69)	Critically Low
Rahman et al. (32)	Critically Low
Rajagopalan & Tammimies (70)	Critically Low
Rezaee (83)	Critically Low
Ribas et al. (62)	Critically Low
Rogers et al. (71)	Low
Salgado et al. (63)	Critically Low
Santana et al. (51)	Critically Low
Senior et al. (38)	Moderate
Silva et al. (39)	Critically Low
Simeoli et al. (72)	Critically Low
Sohn et al. (84)	High
Solek et al. (85)	Low
Song et al. (40)	Low
Swinckels et al. (73)	Critically Low
Taneera & Alhajj (86)	Critically Low
Tian et al. (74)	Low
Toki (75)	Critically Low
Uddin et al. (76)	Critically Low
Valliani et al. (30)	Critically Low
Vimbi et al. (87)	Critically Low
Wei et al. (6)	Critically Low
Welch et al. (52)	Critically Low
Wen et al. (77)	Critically Low
Zaheer & Akhtar (88)	Critically Low
Zhang et al. (41)	Critically Low
Zhang-James et al. (64)	Critically Low

A detailed breakdown of the critical domains (see **Supplementary Figure 1**) reveals systematic deficiencies in transparency and rigor. The most prevalent flaw was the lack of a pre-registered protocol (Item 2), with 81.3% of the reviews failing to establish explicit methods prior to conduct, which significantly increases the risk of selective reporting. Furthermore, transparency regarding study selection was notably compromised: 73.4% of the reviews did not provide a list of excluded studies with justifications (Item 7), a key requirement for reproducibility.

Regarding the assessment of evidence, 65.6% of the authors failed to use a satisfactory technique to assess the Risk of Bias (RoB) in individual studies (Item 9), and consequently, 82.8% did not account for this risk when interpreting or discussing their results (Item 13). While search strategies (Item 4) showed slightly better performance, only 7.8% were comprehensive, with the majority (54.7%) rated as ‘Partial’ due to the omission of grey literature or trial registries. Finally, statistical combination and publication bias impact items (11 and 15, respectively) were largely inapplicable (N/A > 87%) given the qualitative nature of most of the included reviews. This quality assessment underscores that the impact of AI on neurodevelopmental disorders is currently constrained by procedural rigor. The prevalence of failures in protocol registration (81.3%) and lack of transparency in selection (73.4%) indicate that the field must prioritize methodological robustness over merely increasing algorithmic accuracy.

## 4 Discussion

This umbrella review is the first to synthesize the current state of the art on the use of AI in the diagnosis of neurodevelopmental disorders during childhood, evaluating advances, challenges, limitations, and potential integration in real clinical contexts. In this umbrella review, 64 systematic reviews and meta-analyses were examined to obtain a comprehensive and accurate overview of the body of evidence to date.

The findings revealed notable progress in the development and application of AI-based models, particularly regarding the diagnosis of ASD and ADHD. In this respect, the combination of machine learning algorithms, deep learning techniques, and multimodal data fusion (integrating information from diverse sources such as neuroimaging, audio recordings, behavioral metrics, and clinical questionnaires) has been shown to achieve high levels of diagnostic accuracy. Nevertheless, as indicated by Iyortsuun et al. (58) and Ding et al. (9), considerable methodological heterogeneity, variability in study designs, and a lack of extensive clinical validation constitute major obstacles that hinder the immediate translation of these advances into healthcare practice. Additionally, most systematic reviews and meta-analyses examined in this umbrella review showed critically low methodological quality, and although the most rigorous studies focused on ASD and ADHD, further research is warranted to inform scientifically and clinically relevant decisions on these neurodevelopmental conditions.

Despite the predominance of supervised learning models, such as Support Vector Machines (SVM) and Convolutional Neural Networks (CNN), in neuroimaging and EEG signal analysis, there is a critical need to evaluate architecture selection and hyperparameter optimization, aspects often overlooked in the reviewed literature. The application of unsupervised learning paradigms, such as clustering or principal component analysis (PCA), has emerged as a necessary alternative for identifying biotypes within the symptomatologic heterogeneity of NDDs. However, the computational efficiency of these models remains an underreported challenge that affects their viability in real-time clinical settings.

Furthermore, AI-assisted diagnosis faces nonlinear challenges inherent in high-dimensional data, such as those obtained through fMRI or motion sensors. To address these complexities, it is essential to integrate state-of-the-art methods that overcome the limitations of traditional approaches (93). In this regard, the incorporation of Fourier attention mechanisms, specifically frequency-channel attention factorization (94) and wavelet attention models (95, 96), offers significant potential for capturing complex spatiotemporal dependencies in neurophysiological biomarker data. These advanced architectures allow for a more robust decomposition of the signal features, strengthening the models against noise and biological variability.

In response to these limitations, the field is witnessing rapid evolution toward the use of generative AI. Large-scale language models (LLMs), such as MedGemma, have demonstrated an increasing ability to reason through complex medical texts and clinical records (84). At the same time, Generative Adversarial Networks (GANs) have emerged as essential tools for mitigating data scarcity through synthetic sample augmentation (97). In this context, the efficacy of advanced architectures such as the F-GAN-NTD model, recently proposed by Wang et al. (98), stands out. This model fuses generative networks with non-negative tensor decomposition theory to extract nonlinear features from complex data (such as fMRI), demonstrating significant improvements in the classification and restoration of incomplete data compared to traditional approaches. Recent research suggests that these technologies not only support diagnosis but also enable a deeper understanding of the underlying pathophysiological mechanisms.

At the same time, it is essential to acknowledge the current limitations in the search for biomarkers with sufficient sensitivity and specificity to support the diagnosis of the diverse neurodevelopmental disorders, as noted by Srivastava et al. (99)Hanly et al. (100), and Cortese et al. (101). In this sense, scientific consensus indicates that, to date, there are no biomarkers available that permit the replacement of a specialized evaluation by a clinician (clinical psychologist or psychiatrist), which still remains essential to confirm the diagnosis and ensure the validity of future clinical decisions. This issue is particularly relevant in the case of ASD, where neuroimaging—one of the data sources frequently analyzed by AI in diagnostic studies—while offering promising advances, continues to present clear limitations. Specifically, recent literature, such as the review by Schielen et al. (102), underscores that factors, including sample heterogeneity, limited generalizability of models, and moderate accuracy of neuroimaging-based approaches, prevent this technology from reliably replacing specialized clinical judgement on its own. Consequently, the integration of AI-based tools should be regarded as a valuable complement, but not a substitute, for interdisciplinary clinical assessment.

With respect to another disorder in which AI demonstrated greater diagnostic efficacy in this umbrella review, ADHD, it has been observed that this diagnosis presents particular challenges when it co-occurs with other neurodevelopmental disorders. In this regard, Gionet et al. (103) highlighted the specific difficulty in diagnosing ADHD when it is comorbid with epilepsy. Likewise, observational studies, such as that of Perera et al. (104), emphasize the additional diagnostic challenges in cases of comorbidity with intellectual disabilities. These findings suggest the need to determine the extent to which AI models can contribute to overcoming such complex diagnostic barriers, thereby improving both the precision and speed of comorbidity identification.

As mentioned earlier, the main challenges that have commonly complicated the diagnosis of neurodevelopmental disorders include: (1) the overlap of symptoms across different clinical conditions (4–6); (2) the high comorbidity that obscures diagnostic boundaries (5, 8); and (3) heterogeneity in symptom progression and response to interventions (4, 5). In this sense, AI models have demonstrated significant potential to partially address these limitations, for example, through algorithms capable of identifying complex patterns in heterogeneous datasets or integrating clinical, neurocognitive, and behavioral variables within a single predictive model. However, assuming that such AI technologies will provide a definitive solution to address these challenges would risk falling into an optimism bias. It is, therefore, necessary to evaluate the robustness of AI models across large, diverse, and representative populations, as well as to address requirements concerning transparency, interpretability, and the ethical considerations indispensable for its responsible implementation (28, 60, 105). Interestingly, AI models may be very effective and efficient when researching neurodevelopmental disorders from a dimensional, and not a mere categorical, perspective (i.e., DSM-5-TR or ICD-11 classifications), by comprehensively integrating multimodal data (genetic/molecular, neuroimaging, electrophysiological, neuropsychological, and behavioral) (106).

Overall, these results provide evidence of a ‘performance paradox’ due to the gap between the technical excellence of AI models and their lack of real-world impact, which warrants further examination given its implications for both clinical practice and future research directions. On the one hand, from a clinical perspective, this paradox in AI models suggests that clinicians should interpret AI-assisted diagnostic outputs cautiously, recognizing that high accuracy reported in research may not translate directly to their patient populations, particularly those with atypical presentations, comorbidities, or from underrepresented demographic groups. Therefore, AI tools should be positioned as decision-support systems requiring clinician oversight rather than autonomous diagnostic instruments (107). On the other hand, from a research perspective, this paradox highlights the urgent need to shift evaluation paradigms from internal accuracy metrics toward external validity indicators, including performance across multiple sites, stability over time, and consistency across demographic subgroups (108). Future studies should prioritize reporting not only sensitivity and specificity but also calibration metrics, subgroup analyses, and failure mode characterization. Addressing this paradox is essential for building clinician trust and achieving the meaningful integration of AI models into neurodevelopmental assessment workflows.

Consequently, the findings of this umbrella review suggest that AI should be understood as a complementary rather than a substitute tool in the clinical diagnostic process. Its genuine adoption in clinical contexts will depend on overcoming the identified methodological challenges, implementing standardized protocols, ensuring the external validity of models, and promoting regulatory frameworks that address not only data protection but also the explainability of algorithmic decisions. Only through such a balanced approach, grounded in empirical evidence and a clear strategic vision, will it be possible to effectively integrate AI into the diagnosis of neurodevelopmental disorders in real-world clinical practice.

### 4.1 Limitations

Although we have discussed the limitations found in the studies included, it is also relevant to highlight the main limitations of this umbrella review, given that, despite efforts to systematize the analysis, and partly because of this, the following points should be considered:

- A possible bias was due to the exclusion of non-indexed literature, because this umbrella review exclusively included systematic reviews and meta-analyses indexed in bibliographic databases. Therefore, potentially recent and relevant non-indexed studies were not examined. However, this umbrella review did not impose language restrictions to minimize publication bias, and it included three studies in languages other than English (Chinese and Spanish), but the exclusion of non-indexed literature could have filtered out other relevant regional studies.
- Heterogeneity in systematic reviews and meta-analyses included regarding populations, AI models, and diverse outcomes, which complicated the synthesis and interpretation of the findings (92, 109, 110).
- Overlapping primary studies across systematic reviews lead to double counting of data and potential bias in summary estimates. Therefore, methods for handling overlap are often incompletely reported or inconsistently applied. We acknowledge that the complete elimination of overlap is not feasible in umbrella reviews; however, the approach followed in this umbrella review aligns with established methodological guidance (111, 112) and ensures transparent reporting of this limitation.
- Assessment of methodological quality: The use of standardized tools to measure the methodological quality of studies, owing to the lack of an appropriate gold standard, may over-report quality flaws (54). In this sense, for example, in this umbrella review, only 12 studies (18.8%) contained an explicit statement about prior protocols for systematic reviews/meta-analyses, and because item 2 is critical, the corresponding study’s quality was considered, at least, as low, although the remaining items were satisfactory. However, these findings should be interpreted with the understanding that AMSTAR-2 was originally designed for intervention reviews and may impose standards that are challenging to meet in diagnostic accuracy studies, particularly in emerging technological fields. Nevertheless, the prevalence of methodological gaps underscores the need for improved reporting practices as this field matures further.
- Interpretation of performance metrics and model robustness: Although accuracy levels of up to 99% have been reported for certain modalities, these results should be interpreted with caution. In particular, twenty-three percent of the included studies explicitly warned of the risks of overfitting and dataset biases. Furthermore, our analysis revealed a widespread lack of model robustness assessments in the current literature. Most studies have focused on internal performance without validating consistency across diverse populations or external centers. Consequently, we strongly advise that future research establishes model robustness as a mandatory evaluation dimension to distinguish between genuine clinical utility and mere memorization of patterns in limited datasets.

## 5 Conclusion

This umbrella review confirmed a growing interest in how artificial intelligence can support and contribute to identifying biomarkers and diagnosing neurodevelopmental disorders.

In particular, there is considerable potential for facilitating and speeding up the diagnosis of neurodevelopmental disorders using AI models, with especially promising results for ASD, which has been the neurodevelopmental disorder with the highest number of studies investigating diagnostic possibilities to date. Moreover, this disorder has the highest levels of efficacy globally using diverse AI models, such as machine learning, deep learning, and transformers.

However, we should not overlook that the implementation of AI models for an effective clinical activity requires overcoming methodological, ethical, and regulatory challenges that persist in the current scientific literature (9, 58), which is critical to advance from proof-of-concept research to real clinical utility.

To achieve clinical translation, AI models must progress from proof-of-concept demonstrations to standardized implementation protocols through a structured translational pathway as follows:

### 5.1 Multicenter prospective validation

Future studies should prioritize external validation across geographically and demographically diverse sites, employing prospective designs that reflect real-world clinical workflows rather than retrospective convenience samples. Collins et al. (108) note that in order to improve such external validation, it must be performed “on datasets that are representative of the target populations intended for model implementation”, recognizing that variations in healthcare provision, patient demographics, and local practices will naturally affect model performance across settings. Importantly, using existing data that are “merely conveniently available” provides limited and often misleading information on true predictive accuracy.

### 5.2 Open and standardized datasets

The establishment of publicly accessible, well-curated benchmark datasets for neurodevelopmental disorders—analogous to initiatives in other medical imaging domains—would enable direct comparison of AI models and accelerate methodological progress. Current literature highlights that the use of private datasets impedes reproducible research and makes model comparison difficult, recommending standardized protocols and common data repositories (70). Successful examples from the field demonstrate the feasibility of this approach: the ABIDE consortium provides multicenter fMRI data that has become the cornerstone for most machine learning studies in ASD (113, 114), while harmonized neuroimaging cohorts have enabled the generation of normative brain structure curves across the lifespan (115). The usefulness of the data will depend on how complete and robust it is, as well as on the use of similar measures across all samples (106). Reproducible frameworks such as NeuroMark further demonstrate how standardized functional and structural templates enable valid comparisons across disorders and datasets (116, 117).

### 5.3 Standardized reporting guidelines

The development and adoption of AI-specific reporting standards for diagnostic studies on neurodevelopmental disorders, building upon existing frameworks such as STARD-AI (118) and TRIPOD-AI (119), would improve transparency and facilitate meta-analytic synthesis.

### 5.4 Regulatory certification

Engagement with regulatory bodies (e.g., FDA, EMA) to establish clear pathways for AI diagnostic tool approval, including the requirements for continuous performance monitoring post-deployment.

### 5.5 Clinician-AI collaboration frameworks

Design of human-in-the-loop systems where AI outputs serve as decision support, accompanied by explainability features that allow clinicians to understand and verify algorithmic recommendations. As the DECIDE-AI steering group (107) emphasizes, AI-based clinical systems should focus on their “potential to augment rather than replace human intelligence,” recognizing that clinicians remain accountable for their decisions and cannot be expected to follow all algorithmic recommendations without understanding their basis.

In conclusion, this constantly evolving technology could help clinicians increase the accuracy and efficiency of the diagnostic process for neurodevelopmental disorders, for which early detection is fundamental to optimizing clinical prognosis and effectively improving the quality of life of patients.

## Data Availability

The original contributions presented in the study are included in the article/**Supplementary Material**. Further inquiries can be directed to the corresponding author.
