# Supplementary figures and images for: Artificial intelligence support for diagnosis of neurodevelopmental disorders during childhood: an umbrella review
Source: Front Psychiatry. 2026 Mar 18;17:1697185. doi: 10.3389/fpsyt.2026.1697185 (PMC13039104; doi:10.3389/fpsyt.2026.1697185)

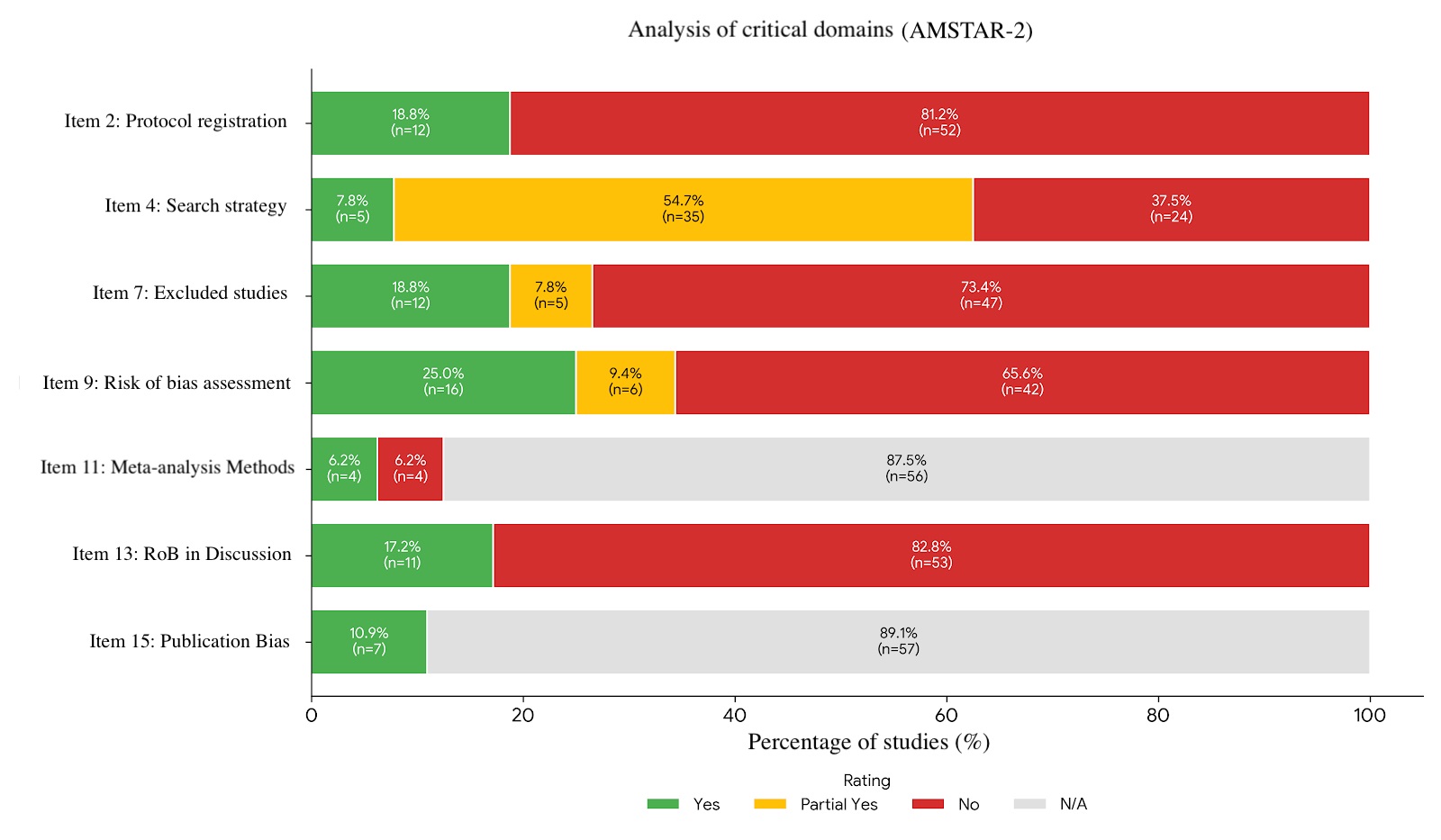

Supplement: Supplementary file 1 [file Image1.jpeg]
