# Supplementary material for: Artificial intelligence support for diagnosis of neurodevelopmental disorders during childhood: an umbrella review
Source: Front Psychiatry. 2026 Mar 18;17:1697185. doi: 10.3389/fpsyt.2026.1697185 (PMC13039104; doi:10.3389/fpsyt.2026.1697185)
Supplement: Supplementary file 2 [file Table1.docx]

**Supplementary material**

Table 1. Data Extraction

| **Covidence No.** | **Study** | **Article title** |  | **Authors** | **Publication**  **year** | **Authors' country of affiliation** | **Artificial intelligence model(s) applied** | **Disorders analyzed** | **Does it analyze levels of efficacy of the model?** | **Conclusions on model confidence levels** | **Overall conclusion of each study** |
| --- | --- | --- | --- | --- | --- | --- | --- | --- | --- | --- | --- |
| **#46** | Alam 2022 | Investigation of Machine Learning Methods for Early Prediction of Neurodevelopmental Disorders in Children |  | Alam, S; Raja, P; Gulzar, Y | 2022 | Saudi Arabia, India | Machine learning (SVM, Random Forest, KNN, Decision Trees, etc.) | Neurodevelopmental disorders in general (ASD, ADHD, Developmental Delay) | Yes, performance metrics are presented: accuracy, sensitivity, specificity, AUC. | Models achieve >90% accuracy in some cases, although with limitations in sample size and data diversity. | Study shows that ML models can support early diagnosis of NDD in children, but larger and more generalizable databases are needed for clinical application. |
| **#63** | Alharthi 2023 | Do it the transformer way: A comprehensive review of brain and vision transformers for autism spectrum disorder diagnosis and classification |  | Alharthi, AG; Alzahrani, SM | 2023 | India | Transformers (Vision Transformers, Brain Transformers, BERT, ViT, Swin, etc.) | Autism Spectrum Disorder (ASD) | Yes. Accuracy, sensitivity, specificity, AUC, etc. are analyzed. | Transformer models show high performance, but require large volumes of data and present computational challenges. | The article highlights the potential of transformer models in automated ASD diagnosis, suggesting their superiority over traditional methods if their technical limitations are addressed. |
| **#53** | Alqaysi 2022 | Diagnosis-Based Hybridization of Multimedical Tests and Sociodemographic Characteristics of Autism Spectrum Disorder Using Artificial Intelligence and Machine Learning Techniques: A Systematic Review |  | Alqaysi, ME; Albahri, AS; Hamid, RA | 2022 | United Kingdom, Saudi Arabia, Jordan, Iraq | Multiple ML and DL models (SVM, Random Forest, CNN, deep networks, KNN, ANN, etc.) combining clinical and sociodemographic data | Autism Spectrum Disorder (ASD) Yes. | Yes, accuracy, sensitivity, specificity, AUC, F1 score, etc. are reported. | Hybrid models show high levels of accuracy, especially with multiple data sources; some exceed 95% accuracy. | Study highlights effectiveness of hybrid approaches combining medical and socio-demographic data using ML to improve ASD diagnosis; further external validation and in diverse samples required |
| **#98** | Alves 2022 | Trends, Limits, and Challenges of Computer Technologies in Attention Deficit Hyperactivity Disorder Diagnosis and Treatment |  | Alves, RMB; da Silva, MNF; Schmitz, EA; Alencar, AJ | 2023 | Spain | Machine learning, neural networks, classification algorithms, expert systems s | Attention- Deficit/ Hyperactivity Disorder (ADHD) | Yes. Accuracy and performance of several models is reported. | Models show high potential with good levels of accuracy, but there are important methodological limitations. | Computational technologies offer promising support for the diagnosis and treatment of ADHD, although there are ethical and technical challenges to be resolved. |
| **#56** | Banos 2024 | Sensing technologies and machine learning methods for emotion recognition in autism: Systematic review |  | Banos, O; Comas-González, Z; Medina, J; Polo-Rodríguez, A; Gil, D; Peral, J; Amador, S; Villalonga, | 2024 | Spain | Machine Learning: SVM, Decision Trees, Random Forest, k-NN, ANN; physiological sensors, computer vision, EEG, etc. | Autism Spectrum Disorder (ASD) | Yes. Reports metrics such as accuracy, sensitivity, specificity. | Most models show promising results, although methodological heterogeneity limits comparability and confidence. | The article concludes that sensor- and ML-based systems show promise for recognizing emotions in people with ASD but urges further standardization and validation in clinical settings. |
| **#120** | Berchio 2025 | EEG Microstates in the Study of Attention-Deficit Hyperactivity Disorder |  | Berchio, C; Kumar, SS; Narzisi, A; Fabbri-Destro, M | 2025 | Spain | No AI model as such is used; EEG microstate analysis is used to characterize patterns | Attention Deficit Hyperactivity Disorder (ADHD) | No. No predictive models are used and no classification metrics are reported. | Not applicable, as no AI models with confidence measures are evaluated. | The article explores the utility of EEG microstats as a tool for understanding ADHD, suggesting that they may offer useful information for future clinical and AI applications. |
| **#39** | Bouchouras and Kotis 2025 | Integrating Artificial Intelligence, Internet of Things, and Sensor-Based Technologies: A Systematic Review of Methodologies in Autism Spectrum Disorder Detection |  | Bouchouras, G; Kotis, K | 2025 | Tunisia | Multiple ML/DL models: SVM, neural networks, CNN, Random Forest, kNN, etc., integrated with sensors and IoT. | Autism Spectrum Disorder (ASD) | Yes. Performance metrics such as accuracy, sensitivity and AUC are reported. | Models combined with IoT show high performance in controlled tests, but limitations in their application in real-world environments. | The article concludes that the integration of AI with IoT technologies has great potential in ASD detection, although more standardization, field validation and attention to ethical issues are required. |
| **#101** | Rogers et al 2024 | Voice as a Biomarker of Pediatric Health: A Scoping Review |  | Bridge2AI Voice Consortium; Rogers, HP; Hseu, A; Kim, J; Silberholz, E; Jo, S; Dorste, A; Jenkins, K | 2024 | United States | Machine learning (Random Forest, SVM, neural networks, CNN, etc.) applied to speech analytics | Multiple pediatric conditions, including ASD, ADHD, language disorders and mental health. | Yes, metrics such as accuracy, sensitivity, specificity are presented. | Models show good accuracy on some tasks, but confidence is limited by methodological variability and paucity of data. | The article highlights the potential of AI speech analysis as a non-invasive biomarker in pediatric health but urges further standardization and external validation. |
| **#31** | Cavus 2021 | A Systematic Literature Review on the Application of Machine-Learning Models in Behavioral Assessment of Autism Spectrum Disorder |  | Cavus, N; Lawan, AA; Ibrahim, Z; Dahiru, A; Tahir, S; Abdulrazak, UI; Hussaini, A | 2021 | Pakistan | Machine Learning: SVM, Decision Tree, Random Forest, ANN, CNN, k-NN, etc. | Autism Spectrum Disorder (ASD) | Yes, metrics such as accuracy, sensitivity, specificity, F1 score, etc. are analyzed. | Some models exceed 90% accuracy, but limitations in sample size and lack of standardization are identified. | The article concludes that ML in behavioral assessment of ASD offers great potential, but needs common protocols, more validated data and external validation for clinical use. |
| **#23** | Cerasuolo 2025 | The Potential of Virtual Reality to Improve Diagnostic Assessment by Boosting Autism Spectrum Disorder Traits |  | Cerasuolo, M; De Marco, S; Nappo, R; Simeoli, R; Rega, A | 2024 | Switzerland, Italy, Australia, Netherlands | Not directly applicable to AI, but future integration in virtual reality (VR) environments is suggested. | Autism Spectrum Disorder (ASD) | No. AI models and predictive metrics are not evaluated. | Not applicable in this context | The article explores how VR can induce behaviors indicative of ASD in controlled contexts, improving clinical assessment, and proposes future integration of AI for more objective analyses. |
| **#99** | Chen 2023 | Evaluation of Risk of Bias in Neuroimaging-Based Artificial Intelligence Models for Psychiatric Diagnosis |  | Chen, ZY; Liu, XR; Yang, QW; Wang, YJ; Miao, K; Gong, Z; Yu, Y; Leonov, A; Liu, CL; Feng, ZZ; Chuan-Peng, H | 2023 | Canada, USA | Machine learning and deep learning applied to neuroimaging (not all models specified) | Psychiatric disorders (schizophrenia, mood disorders, etc.) Yes. | Yes, they assess accuracy and bias using standardized tools such as PROBAST. | High risk of methodological bias in most of the reviewed studies, despite apparently high metrics. | The study warns about the uncritical use of AI in psychiatric neuroimaging, recommending to improve methodological quality and transparency in reporting. |
| **#84** | Cruz 2023 | Application of Machine Learning in Learning Problems and Disorders: A Systematic Review |  | Cruz, MA; Hurtado, OAC; Madariaga, EC | 2023 | Peru | Machine Learning and Deep Learning: SVM, k-NN, Naive Bayes, Decision Tree, MLP, CNN, etc. | Learning Disorders: ADHD, dyslexia, dysgraphia, etc. | Yes, metrics such as accuracy, sensitivity, F1, AUROC, among others, are reported. | Models achieve good performance, with some techniques exceeding 85% accuracy; however, the need for standardization is cautioned. | The article shows that ML shows promise in early diagnosis and classification of learning disabilities, although common protocols and validation in real-world settings are required. |
| **#20** | Das 2023 | Machine Learning Approaches for Electroencephalography and Magnetoencephalography Analyses in Autism Spectrum Disorder: A Systematic Review |  | Das S; Zomorrodi R; Mirjalili M; Kirkovski M; Blumberger DM; Rajji TK; Desarkar P | 2023 | United Kingdom, Australia | Machine Learning: SVM, Random Forest, k-NN, ANN, CNN, etc. applied to EEG and MEG | Autism Spectrum Disorder (ASD) | Yes. Metrics such as accuracy, sensitivity, specificity, F1, AUC are included. | Models achieve accuracy between 70% and 95%, but limitations in sample size, data heterogeneity and cross-validation are identified. | The article concludes that ML applied to EEG/MEG is promising for ASD diagnosis, but requires more standardized protocols, larger samples and rigorous clinical evaluation. |
| **#76** | by Barros 2023 | Computer-aided diagnosis of neurodevelopmental disorders and genetic syndromes based on facial images - A systematic literature review |  | de Barros, FRD; da Silva, CNF; Michelassi, GD; Brentani, H; Nunes, FLS; Machado-Lima, A | 2023 | Portugal | Machine Learning and Deep Learning: CNN, autoencoders, Random Forest, SVM, etc. applied to facial image analysis. | Neurodevelopmental disorders and genetic syndromes (e.g. ASD, Down's syndrome, Williams, etc.) | Yes, accuracy, sensitivity, specificity, AUC are reported. | Several models show high performance (>90% in some cases), but most lack extensive clinical validation. | The article concludes that AI applied to facial imaging is promising to support diagnosis of neurogenetic disorders, but more validation in real clinical settings and more diversity in training data are needed. |
| **#115** | Devi 2025 | The emergence of artificial intelligence in autism spectrum disorder research |  | Devi, KBI; Vincent, PMDR | 2025 | India | Multiple models: SVM, neural networks, random forest, CNN, deep learning | Autism Spectrum Disorder (ASD) | Yes. Presents metrics such as accuracy, sensitivity and specificity in the studies reviewed. | Variability in confidence; highlights the need for further validation and replication in real clinical settings. | The article underlines the growing role of AI in ASD research, highlighting its potential for diagnosis and intervention, but warns of limitations in generalizability and ethics. |
| **#22** | Ding 2024 | Deep learning approach to predict autism spectrum disorder: a systematic review and meta-analysis |  | Ding Y; Zhang H; Qiu T | 2024 | China | Deep Learning: CNN, DNN, RNN, BLSTM, VGG19, ResNet50V2, EfficientNet, MobileNet, Xception, etc. | Autism Spectrum Disorder (ASD) | Yes. Meta-analysis of sensitivity (0.95), specificity (0.93) and AUC (0.98). | High confidence with consistent results in different studies, although with high heterogeneity. | The DL technique shows high sensitivity and specificity for classifying ASD. Further research is recommended to standardize models and validate their clinical utility. |
| **#88** | Dwyer and Koutsouleris 2022 | Annual Research Review: Translational machine learning for child and adolescent psychiatry |  | Dwyer, D; Koutsouleris, N | 2022 | Germany, Norway, UK, Ireland, USA. | Traditional machine learning and deep learning, incl. SVM, Random Forest, CNN, multivariate analysis | Child and adolescent psychiatric disorders (depression, anxiety, ADHD etc.) Yes. | Yes, model results are discussed in terms of accuracy, replicability and clinical validity. | Although some models are promising, there is a significant gap between research accuracy and reliable clinical application. | The article argues for a translational approach integrating AI in child psychiatry with emphasis on reproducibility, generalizability and ethics. |
| **#71** | Fatima 2024 | Machine learning approaches for neurological disease prediction: A systematic review |  | Fatima, A; Masood, S | 2024 | India | Machine learning: Decision Tree, Random Forest, SVM, k-NN, Naive Bayes, ANN, among others. | Neurological diseases (including Alzheimer's, Parkinson's, epilepsy, ASD, ADHD) | Yes. Includes metrics such as accuracy, sensitivity, specificity, AUC. | Models have high performance, especially Random Forest and SVM, although confidence depends on quality and quantity of data. | The article concludes that ML is useful for predicting neurological diseases, but recommends the use of large, validated and standardized datasets for better clinical outcomes. |
| **#133** | Francese and Yang 2022 | Supporting autism spectrum disorder screening and intervention with machine learning and wearables: a systematic literature review |  | Francese, R; Yang, XM | 2021 | China | Mainly machine learning (SVM, kNN, Random Forest, neural networks, etc.) | Autism Spectrum Disorder (ASD) | Yes. Metrics such as accuracy, sensitivity, specificity are reported in several studies. | In general, high accuracy in models, but limited external validity and need for validation in real-world settings. | ML models combined with wearable sensors show potential to improve screening and intervention in ASD, although more longitudinal and large sample size studies are needed. |
| **#12** | Fusaroli 2017 | Is Voice a Marker for Autism Spectrum Disorder? A Systematic Review and Meta-Analysis |  | Fusaroli, Riccardo; Lambrechts, Anna; Bang, Dan; Bowler, Dermot M.; Gaigg, Sebastian B. | 2017 | Denmark, United Kingdom | Acoustic speech analysis with statistical techniques and ML (SVM, Naive Bayes, Random Forest). | Autism Spectrum Disorder (ASD) | Yes. Meta-analysis shows mean AUC of 0.88 | Models perform well, but with high heterogeneity between studies and small sample sizes. | Study concludes that voice characteristics may be useful markers for ASD, but methodological standardization and more studies with larger sample sizes are needed. |
| **#55** | Ganggayah 2025 | Accelerating autism spectrum disorder care: A rapid review of data science applications in diagnosis and intervention |  | Ganggayah, MD; Zhao, D; Liew, EJY; Nor, NAM; Paramasivam, T; Lee, YY; Hasan, NIA; Shaharuddin, S | 2025 | United States | Machine learning (SVM, neural networks, Random Forest, etc.), natural language processing, computer vision | Autism Spectrum Disorder (ASD) | Yes. Accuracy and model performance results are mentioned in several studies. | Several models show high accuracy, but confidence depends on validation, data quality and application context. | Data science has potential to accelerate diagnosis and intervention in ASD, but better implementation practices and rigorous evaluation are required. |
| **#19** | Geng 2020 | Autism spectrum disorder risk prediction: A systematic review of behavioral and neural investigations |  | Geng X; Kang X; Wong PCM | 2020 | Italy | Machine Learning: SVM, k-NN, Random Forest, ANN, CNN; behavioral analysis, EEG, fMRI, genetics | Autism Spectrum Disorder (ASD) | Yes. Features metrics such as accuracy, sensitivity, specificity. | Some models show high accuracy (>90%), but with variability according to data type; the need for external validation is stressed. | The article concludes that ASD risk prediction using ML is feasible, especially combining behavioral and neural data, although longitudinal studies and further validation are required. |
| **#96** | Hu 2023 | Applications of Deep Learning to Neurodevelopment in Pediatric Imaging: Achievements and Challenges |  | Hu, MJ; Nardi, C; Zhang, HH; Ang, KK | 2023 | United States | Deep learning (CNNs, autoencoders, GANs, LSTMs, transformers) applied to pediatric neuroimaging | Neurodevelopment in pediatric population (not limited to specific disorders) | Yes. Reports metrics such as accuracy, sensitivity, specificity in specific tasks. | Some models achieve high accuracy, but caution on data bias, pediatric data paucity and lack of generalizability | DL has great potential in pediatric neurodevelopmental imaging, but requires solutions to improve data, interpretability and ethics in clinical use. |
| **#32** | Huda 2024 | Advancements in automated diagnosis of autism spectrum disorder through deep learning and resting-state functional MRI biomarkers |  | Huda, S; Khan, DM; Masroor, K; Warda; Rashid, A; Shabbir, M | 2024 | India | Deep Learning: CNN, autoencoders, ResNet, Graph Convolutional Networks applied to rs-fMRI | Autism Spectrum Disorder (ASD) | Yes, metrics such as accuracy, sensitivity, specificity, AUC are presented. | Models show high accuracy (>90%), although the authors caution about the need for cross-validation and generalization. | The article reviews recent advances in automated diagnosis of ASD with DL on rs-fMRI, highlighting the clinical potential, but emphasizing the need for replication and robust validation. |
| **#50** | Iyortsuun 2023 | A Review of Machine Learning and Deep Learning Approaches on Mental Health Diagnosis |  | Iyortsuun, NK; Kim, SH; Jhon, M; Yang, HJ; Pant, S | 2023 | South Korea | Machine Learning and Deep Learning: SVM, Decision Tree, Naive Bayes, Random Forest, k-NN, ANN, CNN, etc. | Mental disorders: depression, anxiety, bipolar disorder, schizophrenia, etc. | Yes. Presents metrics such as accuracy, sensitivity, specificity, F1, etc. | High levels of accuracy are highlighted, but with limitations due to sample size and lack of cross-validation. | The article concludes that ML and DL are useful for to support the diagnosis of mental illness, but emphasizes the need for more clinical studies with robust validation and standardization of data. |
| **#54** | Joudar 2023 | Artificial intelligence-based approaches for improving the diagnosis, triage, and prioritisation of autism spectrum disorder |  | Joudar, SS; Albahri, AS; Hamid, RA; Zahid, IA; Alqaysi, ME; Albahri, OS; Alamoodi, AH | 2023 | Australia | Machine Learning, Deep Learning, NLP and Recommendation Algorithms | Autism Spectrum Disorder (ASD) | Yes, systems with high sensitivity and specificity for triage and diagnosis are mentioned. | Systems show high potential, but ethical evaluation and clinical validation are needed before widespread implementation. | The article reviews how AI can improve ASD diagnosis and prioritization processes, with promising approaches still in the research and regulatory development phase. |
| **#118** | Khan and Shang 2025 | A short investigation of the effect of the selection of human brain atlases on the performance of ASD's classification models |  | Khan, NA; Shang, XQ | 2025 | China | Multiple models: Graph Neural Networks, CNN, GAN, autoencoders, model assembly, etc. | Autism Spectrum Disorder (ASD) | Yes, reports accuracy results from various models depending on the atlas used. | Results are atlas dependent; dense models such as CC400 tend to be more accurate but require more resources. | The article highlights that brain atlas selection has a crucial impact on the performance of models for classifying ASD; standardization and more research on atlas combination and site validation is recommended. |
| **#102** | Khare 2023 | Application of data fusion for automated detection of children with developmental and mental disorders: A systematic review of the last decade |  | Khare, SK; March, S; Barua, PD; Gadre, VM; Acharya, UR | 2023 | Greece | ML and data fusion techniques: SVM, Random Forest, neural networks, CNN, multi-source combination techniques. | Neurodevelopmental and mental disorders: ASD, ADHD, anxiety, depression, etc. Yes. | Yes, metrics such as accuracy, sensitivity, specificity, etc. are reviewed. | Models combined with data fusion tend to improve accuracy, but still face problems of interoperability and standardization. | The article concludes that data fusion improves the performance of models for detecting disorders in children, although more robust studies with more diverse data and reproducible techniques are needed. |
| **#73** | Kohli 2022 | The Role of Intelligent Technologies in Early Detection of Autism Spectrum Disorder (ASD) |  | Kohli, M; Kar, AK; Sinha, S | 2024 | India | AI and ML in general: not all models are specified, but intelligent systems and automated analysis are discussed. | Autism Spectrum Disorder (ASD) | Not with quantitative data but mentions potential for diagnostic improvement. | Does not yet report concrete metrics of confidence; future validation in clinical settings advocated | Article highlights the growing role of smart technologies in the early detection of ASD, suggesting that combinations of AI and smart devices may revolutionize pediatric diagnostics |
| **#61** | Li 2024 | Identification of autism spectrum disorder based on electroencephalography: A systematic review |  | Li, J; Kong, XL; Sun, LL; Chen, X; Ouyang, GX; Li, XL; Chen, SY | 2024 | Iran | SVM, Random Forest, kNN, neural networks, CNN, LSTM, etc. (applied to EEG signals) | Autism Spectrum Disorder (ASD) | Yes. Metrics such as accuracy, sensitivity, specificity, AUC are analyzed. | Models show high potential, but there is variability in results and limitations in sample size and generalizability. | Concludes that EEG analysis combined with AI models shows promise for early diagnosis of ASD but requires further validation and standardization. |
| **#42** | Rajagopalan and Tammimies 2024 | Predicting neurodevelopmental disorders using machine learning models and electronic health records - status of the field |  | Rajagopalan, SS; Tammimies, K | 2024 | Sweden | Machine learning (Random Forest, logistic regression, boosting, neural networks, etc.) applied to EHRs | Neurodevelopmental disorders (ASD, ADHD, intellectual disability, etc.) Yes. | Yes, it presents performance metrics such as AUC, accuracy, sensitivity. | Some models show good performance, but quality and heterogeneity of EHR data limits confidence in results | The article highlights the potential of EHRs combined with ML to predict TND, but warns about important methodological, ethical and technical challenges. |
| **#66** | Mengi 2022 | A systematic literature review on traditional to artificial intelligence based socio-behavioral disorders diagnosis in India |  | Mengi, M; Malhotra, D | 2022 | India | Artificial neural networks, SVM, Random Forest, kNN, Naive Bayes, CNN, etc. | Socio-behavioral disorders (including ASD, ADHD, intellectual disability, etc.) | Yes. Compares accuracy, sensitivity, specificity of models. | Some models achieve high accuracy (>90%), but lack standardization and large-scale validation. | The article highlights the evolution of traditional methods towards AI-based techniques for diagnosing socio-behavioral disorders in India, recommending standardized assessment frameworks. |
| **#83** | Mengi 2022 | Artificial Intelligence Based Techniques for the Detection of Socio-Behavioral Disorders |  | Mengi, M; Malhotra, D | 2022 | India | AI based on Machine Learning: SVM, Decision Tree, Random Forest, ANN, CNN, etc. | Socio-behavioral disorders s: ASD, ADHD, anxiety, depression, among others. | Yes. performance metrics such as accuracy, sensitivity and specificity are presented. | The models show good performance in different studies, but the need for further cross-validation and robust clinical data is highlighted. | The article concludes that AI techniques show promise for detecting socio-behavioral disorders, although further standardization and validation in real clinical settings is required. |
| **#44** | Miranda 2021 | Systematic Review of Functional MRI Applications for Psychiatric Disease Subtyping |  | Miranda, L; Paul, R; Pütz, B; Koutsouleris, N; Müller-Myhsok, B | 2021 | United States | Machine learning (k-means, hierarchical clustering, neural networks, multivariate analysis, etc.) on fMRI | Psychiatric disorders (ASD, ADHD, schizophrenia, depression, bipolar) Yes. | Yes, although the focus is more on subtyping methods than on direct clinical classification. | Some methods are able to distinguish relevant functional subtypes, but there is poor replicability and lack of consensus on clinical validity. | The article highlights the value of fMRI with ML for psychiatric subtyping, but urges greater standardization, cross-validation and consensus on methodology. |
| **#33** | Moridian 2022 | Automatic autism spectrum disorder detection using artificial intelligence methods with MRI neuroimaging: A review |  | Moridian, P; Ghassemi, N; Jafari, M; Salloum-Asfar, S; Sadeghi, D; Khodatars, M; Shoeibi, A; Khosravi, A; Ling, SH; Subasi, A; Alizadehsani, R; Gorriz, JM; Abdulla, SA; Acharya, UR | 2022 | Turkey | Deep learning and ML: CNN, autoencoders, SVM, Random Forest, LSTM, etc., on MRI data (structural and functional) | Autism Spectrum Disorder (ASD) | Yes. Metrics such as accuracy, sensitivity, specificity ad, F1 score, etc. are presented. | Some models achieve accuracy higher than 95%, although the lack of multicenter studies and problems of overfitting are highlighted. | Concludes that AI on neuroimaging has great potential in the detection of ASD, but requires robust clinical validation, more diverse data and ethical approach in model development. |
| **#40** | Parlett-Pelleriti 2023 | Applications of Unsupervised Machine Learning in Autism Spectrum Disorder Research |  | Parlett-Pelleriti, CM; Stevens, E; Dixon, D; Linstead, EJ | 2023 | Turkey | Unsupervised learning (hierarchical clustering, k-means, t-SNE, UMAP, PCA, etc.) | Autism Spectrum Disorder (ASD) | Not in classical terms (accuracy/sensitivity), but they do assess cluster coherence and validity. | Relevant patterns are observed that may reflect phenotypic subtypes; but need for external validation is noted. | Article highlights how unsupervised learning can help identify subgroups within the autism spectrum, offering a complementary route to traditional diagnosis |
| **#129** | Pereira-Sanchez and Caste llanos 2021 | Neuroimaging in attention-deficit/hype ractivity disorder |  | Pereira-Sanchez, V; Castell anos, FX | 2021 | United States, Spain | Studies with machine learning on neuroimaging, including CNN, SVM, Random Forest and multivariate models are discussed. | Attention Deficit Hyperactivity Disorder (ADHD) Yes. Studies are discussed for accuracy and precision. | Yes. Studies with accuracy of up to 85% are discussed, although questioning validity due to methodological problems. | Results report high accuracies but are inflated by methodological biases such as overfitting and lack of external validation. | Although neuroimaging in ADHD is not yet clinically applicable, advances in multimodal integration and open science are recognized; greater robustness and replicability of methods needed |
| **#75** | Quaak 2021 | Deep learning applications for the classification of psychiatric disorders using neuroimaging data: Systematic review and meta-analysis |  | Quaak, M; van de Mortel, L; Thomas, RM; van Wingen, G | 2021 | Germany, Switzerland | Deep learning (CNNs, autoencoders, LSTM, etc.) on neuroimaging data | Psychiatric disorders (including ASD, ADHD, schizophrenia, depression, etc.) Yes. | Yes, including meta-analyses with measures such as accuracy, AUC and sensitivity. | Moderate-high average overall accuracy, although with high heterogeneity between studies and possible publication biases. | Concludes that deep learning applied to neuroimaging is promising in psychiatry, but needs further standardization and external validation. |
| **#58** | Quintero 2024 | Predicting ADHD with Machine Learning: Systematic Literature Review |  | Quintero, C, Gil, V; Cerpa, RM; Herrera, M | 2024 | Colombia | Machine Learning: SVM, Random Forest, k-NN, decision trees, neural networks es, among others. | Attention Deficit Hyperactivity Disorder (ADHD). | Yes. Presents metrics such as accuracy, sensitivity, specificity, F1 | Several models exceed 85% accuracy, although it highlights the need for more studies with real, validated clinical data. | The article concludes that ML is promising for predicting ADHD, but there are still important challenges in data standardization, interpretability and external validation. |
| **#30** | Rezaee 2025 | Machine learning in automated diagnosis of autism spectrum disorder: a comprehensive review |  | Rezaee, K | 2025 | Iran | SVM, CNN, image analysis, EEG signals, eye tracking, voice analysis, among others. | Autism Spectrum Disorder (ASD) | Yes. Analyze accuracy, sensitivity, computational complexity and clinical applicability. | High accuracy reported in several methods; however, many techniques have limitations in clinical applicability and data requirements. | The article concludes that ML-based techniques have great potential for automated ASD diagnosis, but need improvements in scalability, interpretability and data diversity. |
| **#29** | Ribas 2023 | Technologies to support the diagnosis and/or treatment of neurodevelopmental disorders: A systematic review |  | Ribas, MO; Micai, M; Caruso, A; Fulceri, F; Fazio, M; Scattoni, ML | 2023 | Italy | Machine learning, deep learning, neural networks, SVM, etc. (multiple models) | Neurodevelopmental disorders (ASD, ADHD, learning disabilities, etc.) Yes, evaluate effectiveness of multiple models. | Yes. Evaluate efficacy of multiple models with metrics like AUC, accuracy, sensitivity, specificity, etc. | Conclusion variable per study; some achieve high accuracy (up to 100%), but high risk of bias highlighted in many studies. | Technology has great potential to improve diagnosis and treatment of NDD, but research of higher quality is needed. |
| **#116** | Salgado 2023 | Predicting and monitoring ADHD in adults with machine learning algorithms. |  | Salgado, JDO; Díaz, AD; Zuluaga, JJR | 2023 | Spain | Machine Learning Algorithms: SVM, Random Forest, Naive Bayes, k-NN, neural networks, logistic regression | Attention Deficit Hyperactivity Disorder (ADHD) in adults | Yes. Metrics such as accuracy, sensitivity, specificity and F1 score are presented. | Random Forest and SVM showed better confidence levels, with accuracy close to 90%. | The study concludes that ML algorithms are useful tools to support the diagnosis of ADHD in adults, especially Random Forest and SVM, although validation in larger clinical samples is required. |
| **#110** | Santana 2022 | Resting-state fMRI and machine learning for ASD diagnosis: A review |  | Santana CP; de Carvalho EA; Rodrigues ID; Bastos GS; de Souza AD; de Brito LL | 2024 | Brazil | Machine learning and deep learning: SVM, CNN, GCN, Random Forest, MLP, etc. applied to rs-fMRI | Autism Spectrum Disorder (ASD) | Yes, accuracy, sensitivity, specificity, F1 score, AUC are reported. | Some models exceed 90% accuracy, but the need to avoid overfitting and improve generalization is pointed out. | The article concludes that the combination of rs-fMRI with ML/DL is promising for ASD diagnosis, but urges standardization of protocols and validation in real clinical settings. |
| **#18** | Senior 2021 | Prediction models for child and adolescent mental health: A systematic review of methodology and reporting in recent research |  | Senior, M; Fanshawe, T; Fazel, M; Fazel, S | 2021 | United Kingdom | Multiple: logistic regression, decision trees, random forest, SVM, neural networks, etc. | Mental disorders in children and adolescents (depression, anxiety, ADHD, psychosis, etc.) Yes. | Yes, they assess performance with accuracy, sensitivity, specificity, AUC, etc. | Models have potential, but most have a high risk of bias, and little external validation. | Urgent need to improve methodological quality and transparency in child mental health prediction studies; better validation and reporting practices needed. |
| **#68** | Silva 2021 | The future of General Movement Assessment: The role of computer vision and machine learning - A scoping review |  | Silva, N; Zhang, DJ; Kulvicius, T; Gail, A; Barreiros, C; Lindstaedt, S; Kraft, M; Bölte, S; Poustka, L; Nielsen-Saines, K; Wörgötter, F; Einspieler, C; Marschik, PB | 2021 | United States | Machine learning (SVM, Random Forest, CNN, LSTM, etc.), computer vision | Neurodevelopmental disorders (including cerebral palsy, psychomotor retardation, etc.) Yes. | Yes, it includes efficacy metrics in movement classification. | Models with promising results but still lack standardization and extensive clinical validation. | Concludes that ML and computer vision have great potential to automate GMA in neonates, facilitating early detection of TND, although technical and validation challenges remain. |
| **#36** | Simeoli 2024 | Using Machine Learning for Motion Analysis to Early Detect Autism Spectrum Disorder |  | Simeoli, R; Rega, A; Cerasuolo, M; Nappo, R; Marocco, D | 2024 | Brazil | Machine Learning: Decision Tree, SVM, Random Forest, k-NN, Naive Bayes, etc. | Autism Spectrum Disorder (ASD) | Yes. Metrics such as accuracy, sensitivity, specificity, F1 score are reported. | Models show good levels of accuracy in movement analysis, but need further clinical validation. | The study concludes that ML motion analysis may be useful for early detection of ASD, highlighting the importance of standardizing protocols and validating in diverse clinical populations. |
| **#57** | Solek 2025 | The Role of Artificial Intelligence for Early Diagnostic Tools of Autism Spectrum Disorder: A Scoping Review |  | Solek, P; Nurfitri, E; Sahril, I; Prasetya, T; Rizqiamuti, AF; Burhan; Rachmawati, I; Gamayani, U; Rusmil, K; Chandra, LA; Afriandi, I; Gunawan, K | 2025 | Indonesia | General AI and ML: SVM, neural networks, Random Forest, computer vision techniques, speech analysis, etc. | Autism Spectrum Disorder (ASD) | Yes. Review of studies with metrics such as accuracy, sensitivity and specificity. | Some studies report high accuracy (>90%), although the lack of standardization and clinical validation is noted. | The review concludes that AI has great potential for early ASD diagnostic tools, but requires improvement accessibility, standardization, ethical and clinical evaluation. |
| **#65** | Song 2021 | Machine learning with neuroimaging data to identify autism spectrum disorder: A systematic literature review |  | Song, DY; Topriceanu, CC; Ilie-Ablachim, DC; Kinali, M; Bisdas, S | 2021 | South Korea | Machine Learning: SVM, k-NN, Random Forest, CNN, autoencoders applied to fMRI, sMRI, DTI, etc. | Autism Spectrum Disorder (ASD) | Yes. Metrics included: accuracy, sensitivity, specificity, AUC. | Positive results with models exceeding 85% accuracy, but high heterogeneity in design and sample size. | The article concludes that ML models with neuroimaging are promising for ASD diagnosis, although more standardization and extensive clinical validation is needed. |
| **#111** | Swinckels 2024 | The Use of Deep Learning and Machine Learning on Longitudinal Electronic Health Records for the Early Detection and Prevention of Diseases: A Review |  | Swinckels, L; Ennis, FC; Ziesemer, KA; Scheerman, JFM; Bijwaard, H; de Keijzer, A; Bruers, JJ | 2022 | Netherlands | Deep Learning and Machine Learning: neural networks, Random Forest, SVMs, transformers, LSTM applied to EHRs | Diverse diseases, including neuropsychiatric and neurodevelopmental conditions. | Yes. Includes metrics such as AUC, accuracy, sensitivity, etc. | Good levels of accuracy are reported, although with concerns about generalizability and quality of EHRs. | The article concludes that the use of ML/DL in longitudinal electronic records has great potential for early detection, but requires improved quality and standardization of data, as well as clinical validation. |
| **#51** | Taneera and Alhajj 2025 | Diagnosis of autism spectrum disorder: a systematic review of clinical and artificial intelligence methods |  | Taneera, S; Alhajj, R | 2025 | Australia, United Kingdom | SVM, k-NN, Decision Tree, Random Forest, ANN, CNN | Autism Spectrum Disorder (ASD) | Yes. Metrics such as accuracy, sensitivity, specificity, F1, etc. are reported. | Some models reach up to 98% accuracy, but the authors warn of over-fitting and lack of external validation. | The article highlights that AI methods can complement clinical diagnoses of ASD, but that their use should be supported by rigorous validation and further methodological standardization. |
| **#47** | Tian 2024 | Structural and functional MRI-based machine learning techniques for attention-deficit/hyperactivity disorder diagnosis: A systematic review and meta-analysis |  | Tian, L; Zheng, HL; Zhang, K; Qiu, JW; Song, XJ; Li, SW; Zeng, Z; Ran, BS; Deng, X; Cai, JH | 2024 | China | Machine learning (SVM, Random Forest, neural networks, etc.) applied to fMRI and sMRI | Attention Deficit Hyperactivity Disorder (ADHD) | Yes. Metrics such as accuracy, sensitivity, specificity and AUC are reported. | Moderate to high levels of accuracy were found; however, there is heterogeneity between studies and risk of bias. | Concludes that brain imaging-based ML models have diagnostic potential for ADHD, but stronger methodological standards and external validations are required. |
| **#43** | Uddin 2024 | Deep learning with image-based autism spectrum disorder analysis: a systematic review |  | Uddin, MZ; Shahriar, MA; Mahamood, MN; Alnajjar, F; Pramanik, MI; Ahad, MAR | 2024 | Australia | Deep learning: CNN, ResNet, 3D CNN, DNN, LSTM, autoencoders applied to neuroimaging | Autism Spectrum Disorder (ASD) | Yes, metrics such as accuracy, AUC, sensitivity, specificity are reported. | DL models perform well with AUC between 0.83 and 0.98; limited confidence due to heterogeneity in data and lack of standardization. | The study shows that DL has great potential for imaging-based ASD analysis, but requires data standardization, external validation and greater transparency in methodologies. |
| **#15** | Valliani 2019 | Deep Learning and Neurology |  | Valliani AA; Ranti D; Oermann EK | 2019 | United Kingdom, Switzerland | Deep Learning: CNN, RNN, autoencoders, transformers, etc. applied to neuroimaging, language and EEG. | Neurological and psychiatric disorders (including ASD, ADHD, dementia, epilepsy, etc.). | Yes, comments on sensitivity, specificity and cross-validation in several studies. | Variable confidence depending on pathology and method; more complex models have higher risk of overfitting if not adequately controlled. | The article reviews how deep learning is transforming analytics in neurology, emphasizing its diagnostic potential and the ethical and technical challenges for responsible clinical adoption. |
| **#114** | Lee et al 2024 | State-of-the-Art of Machine Learning in Neurodevelopment Disorder: A Systematic Review |  | Wei, LLY; Ibrahim, AAA; Alfred, R | 2024 | Malasya | Machine learning (SVM, Random Forest, Decision Tree, k-NN, etc.) and Deep Learning (CNN, RNN) | Neurodevelopmental disorders (ASD, ADHD, intellectual disability, etc.) Yes. | Yes, metrics such as accuracy, sensitivity, specificity and AUC are reviewed. | Good levels of confidence are reported for many models, although with caveats about lack of standardization and external validation. | The article concludes that ML and DL offer promising tools for TND diagnosis, but more research is needed on interpretability, ethics and generalized validation. |
| **#104** | Welch 2022 | Use of Mobile and Wearable Artificial Intelligence in Child and Adolescent Psychiatry: Scoping Review |  | Welch, V; Wy, TJ; Ligezka, A; Hassett, LC; Croarkin, PE; Athreya, AP; Romanowicz, M | 2022 | Canada | Machine learning (various models: SVM, Random Forest, neural networks, etc.) on mobile devices and wearables | Psychiatric disorders in children and adolescents (ADHD, ASD, depression, anxiety) Yes. | Yes, metrics such as accuracy and sensitivity are reported in some studies. | Confidence in models is variable; further validation in real clinical contexts is required. | Concludes that AI in mobile devices and wearables holds promise for child mental health, but needs further standardization, validation and ethical assessment |
| **#103** | Wen 2024 | Dimensional Neuroimaging Endophenotypes: Neurobiological Representations of Disease Heterogeneity Through Machine Learning |  | Wen, JH; Antoniades, M; Yang, ZJ; Hwang, G; Skampardoni, I; Wang, RG; Davatzikos, C | 2024 | United States | Unsupervised machine learning (clustering, ICA, functional connectivity, multivariate embeddings) applied to neuroimaging. | Diverse psychiatric disorders (ASD, schizophrenia, mood disorders, etc.) | Not directly; focus is on dimensional characterization rather than clinical prediction. | Methods allow identification of consistent dimensional representations, but are not clinically validated for diagnosis. | The article proposes that dimensional ML approaches applied to neuroimaging can reveal endophenotypes useful for understanding heterogeneity in psychiatry, although they do not yet replace traditional diagnostic criteria. |
| **#45** | Zaheer and Akhtar 2025 | Artificial intelligence as a support to diagnose ADHD: an insight of unorthodox approaches-a scoping review |  | Zaheer, A; Akhtar, A | 2025 | Spain | Machine Learning and alternative approaches: fuzzy logic, evolutionary algorithms, neural networks, etc. | Attention Deficit Hyperactivity Disorder (ADHD) | Yes, performance results such as accuracy, sensitivity and specificity are reported in several studies. | Some methods show high potential, but heterogeneity and lack of standardization limit the confidence. | The article proposes that non-traditional AI approaches can complement ADHD diagnosis, although further clinical validation and systematic comparisons between methods are still required. |
| **#49** | Zhang 2021 | Application of Artificial Intelligence in the MRI Classification Task of Human Brain Neurological and Psychiatric Diseases: A Scoping Review |  | Zhang, Z; Li, GF; Xu, Y; Tang, XY | 2021 | Brazil | Deep learning (CNN, autoencoders, transformers, etc.) and traditional models (SVM, Random Forest) on MRI. | Neurological and psychiatric disorders (ASD, ADHD, schizophrenia, Alzheimer, etc.). | Yes, metrics such as accuracy, AUC, F1, specificity and sensitivity are discussed. | Several models achieve high accuracy (>90%), but the authors warn about problems of generalization, sample size, and bias. | The article highlights the potential of AI in MRI classification of brain diseases, but calls for more studies with heterogeneous, reproducible and clinically relevant data. |
| **#14** | Zhang-James 2023 | Machine Learning and MRI-based Diagnostic Models for ADHD: Are We There Yet? |  | Zhang-James Y; Razavi AS; Hoogman M; Franke B; Faraone SV | 2023 | United States | ML with MRI: SVM, Random Forest, neural networks, ensemble learning, among others. | Attention Deficit Hyperactivity Disorder (ADHD) | Yes. Includes metrics such as accuracy, AUC, sensitivity, specificity. | Most models have low generalizable performance; limitations in sample size, publication bias and variability between studies are reported. | The article concludes that current MRI and ML-based models for ADHD are not ready for clinical use, and more work on replication, standardization and sample diversity is needed. |
| **#138** | Toki 2024 | Using Eye-Tracking to Assess Dyslexia: A Systematic Review of Emerging Evidence |  | Toki, E. I. | 2024 | Greece | Machine Learning (Random Forest, CNN and MLP) | Dyslexia | Yes | The integration of machine learning models, mainly convolutional neural networks (CNN), MLP and Random Forests, together with eye-tracking data has been shown to increase classification capability. Improved sensitivity | The integration of machine learning models, mainly convolutional neural networks (CNN), MLP and Random Forests, together with eye-tracking data has been shown to increase classification capability. Improved sensitivity |
| **#142** | Sohn 2025 | Implementation of generative AI for the assessment and treatment of autism spectrum disorders: a scoping review |  | Jun-Seok Sohn, Eojin Lee, Jae-Jin Kim, Hyang-Kyeong Oh, Eunjoo Kim | 2025 | South Korea | Generative Models (included Transformers) | Autism Spectrum Disorder (ASD) | Yes. The article compares GenAI models with traditional methods and with each other, using metrics such as accuracy, F1-score, precision, recall, clinical validity, and caregiver-therapist feedback. | GenAI models show potential but have important limitations. | The use of GenAI in autism diagnosis, treatment and intervention is rapidly expanding and shows promising results in improving early detection, personalization of interventions and support for caregivers.  However, their clinical effectiveness and safety still require rigorous and standardized validation, as well as human supervision, to avoid errors and biases. Ethical and transparent integration of these technologies into clinical practice and further research on their real impacts in the clinical and social world are recommended. |
| **#130** | Rahman 2020 | A Review of Machine Learning Methods of Feature Selection and Classification for Autism Spectrum Disorder |  | Rahman, MM; Usman, OL; Muniyandi, RC; Sahran, S; Mohamed, S; Razak, RA | 2020 | Malasya | Artificial Neural Networks  Support Vector Machines (SVM)  A priori algorithms  Decision trees  Supervised and unsupervised learning methods (e.g. hierarchical clustering, Gaussian mixture models)  Deep Learning  Feature selection techniques: filter, wrapper and embedded methods, LASSO, Random Forest, etc. | Autistic Spectrum Disorder (ASD) / ADHD | Yes. Rationale: The article analyses the efficacy of machine learning models by applying standard evaluation metrics such as accuracy, sensitivity, specificity, cross-validation, ROC curve, and Unweighted Average Recall (UAR). | Machine learning models - some specified in the article - show high levels of accuracy, sensitivity and specificity in previous studies (reporting values above 90% in several cases), although the result depends on the type of data, model and validation used. They note that models with adaptive feature selection and robust methods improve diagnostic reliability, but warn about the need for external validation and control of data bias (imbalance, quality, etc.). | The use of machine learning methods for feature selection and classification of ASD is promising, allowing faster and more accurate diagnoses compared to conventional methods. The importance of choosing attributes well, reducing dimension, using appropriate validation techniques and working with diverse clinical data is highlighted. The article suggests that these technologies can improve early identification and intervention in autism, although challenges remain in terms of generalization, data integration and external validation. |
| **#145** | Kang 2022 | Applications and Performances of Artificial Intelligence in Assessment and Diagnosis of Communication Disorders: A Systematic Review of the Literatures |  | Kang, HW; Kang, JK; Lee, SB; Sim, HS | 2022 | South Korea | Machine Learning (ML): Decision Tree (DT), Feed Forward Neural Network (FFNN), K-Nearest Neighbors (KNN), Support Vector Machines (SVM)  Deep Learning (DL): Convolutional Neural Network (CNN), Deep Neural Networks (DNN), Deep Recurrent Neural Network (DRNNN), Long Short-Term Memory (LSTM), Recurrent Neural Network (RNN) | Language Learning Disability, Dyslexia | Yes, model efficacy levels were analyzed using metrics such as accuracy, sensitivity, specificity, area under the curve (AUC), precision, F1-score and recall, which were reported with different ranges depending on the model and the specific task (e.g., accuracy ranged from 59.38% to 99.23%). | The models show variable results depending on the task and data used, with some achieving similar or superior performance to experts, although there are limitations due to possible problems of overfitting, data bias and lack of independence between reference and prediction in certain studies, so they recommend greater transparency and explainability to reinforce the clinical confidence in their use. | The article concludes that the application n of AI in the assessment and diagnosis of communication disorders is growing steadily, with special interest in acoustic analysis and the pediatric population, highlighting that clinical applicability depends on interdisciplinary work, access to quality data and the integration of language professionals in development, as well as recommending the expansion of the types of analysis to strengthen r confidence and clinical utility of these systems. |
| **148** | Vimbi 2025 | Application of Explainable Artificial Intelligence in Autism Spectrum Disorder Detection |  | Vimbi, V; Shaffi, N; Sadiq, MAK; Sirasanagandla, SR; Aradhya, VNM; Kaiser, MS; Wang, SQ; Mahmud, M | 2025 | Oman, India, Bangladesh, China and Saudi Arabia. | Machine learning models, deep learning and explainable artificial intelligence (XAI) frameworks applied to the prediction of autism spectrum disorder (ASD) are analyzed, including twenty classifiers and five XAI frameworks. | Autism Spectrum Disorder (ASD) | Yes, justified because the review analyses different studies on XAI in ASD prediction models, where benefits (improved transparency, confidence and clinical adoption) and challenges of interpretability vs. accuracy of the models are discussed. | It is concluded that XAI systems increase confidence and transparency in prediction, but the challenge of balancing interpretability and accuracy remains. | The article highlights that explainable artificial intelligence significantly improves confidence and adoption of ASD detection systems in clinical settings, although the challenge of maintaining accuracy without losing interpretability in future models remains. |
