# Supplementary material for: Artificial intelligence support for diagnosis of neurodevelopmental disorders during childhood: an umbrella review
Source: Front Psychiatry. 2026 Mar 18;17:1697185. doi: 10.3389/fpsyt.2026.1697185 (PMC13039104; doi:10.3389/fpsyt.2026.1697185)
Supplement: Supplementary file 3 [file Table2.docx]

**Supplementary material**

Table 2. Excluded studies

| **Title** | **Authors** | **Published Year** | **Notes** |
| --- | --- | --- | --- |
| **Wearables and mobile technologies in autism spectrum disorder interventions: A systematic literature review** | Koumpouros, Yiannis; Kafazis, Theodoros | 2019 | Exclusion reason: No diagnostic tool |
| **Deep learning for neuroimaging-based diagnosis and rehabilitation of Autism Spectrum Disorder: A review** | Khodatars, M; Shoeibi, A; Sadeghi, D; Ghaasemi, N; Jafari, M; Moridian, P; Khadem, A; Alizadehsani, R; Zare, A; Kong, Y; Khosravi, A; Nahavandi, S; Hussain, S; Acharya, UR; Berk, M | 2021 | Exclusion reason: Wrong study design |
| **The emerging role of artificial intelligence in the diagnosis and treatment of autism spectrum disorder and attention-deficit/hyperactivity disorder** | Nasrallah, M; El Moghrabi, A; Kayal, M; Matar, D; Alwan, I; Fakhouy, M | 2025 | Exclusion reason: Wrong study design |
| **AI-based non-invasive imaging technologies for early autism spectrum disorder diagnosis: A short review and future directions** | Abdelrahim, M; Khudri, M; Elnakib, A; Shehata, M; Weafer, K; Khalil, A; Saleh, GA; Batouty, NM; Ghazal, M; Contractor, S; Barnes, G; El-Baz, A | 2025 | Exclusion reason: Wrong study design |
| **Machine Learning Methods for Diagnosing Autism Spectrum Disorder and Attention- Deficit/Hyperactivity Disorder Using Functional and Structural MRI: A Survey** | Eslami, T; Almuqhim, F; Raiker, JS; Saeed, F | 2021 | Exclusion reason: Wrong study design |
| **Eye Tracking Biomarkers for Autism Spectrum Disorder Detection using Machine Learning and Deep Learning Techniques: Review** | Jeyarani, RA; Senthilkumar, R | 2023 | Exclusion reason: Wrong study design |
| **Food for Thought: Machine Learning in Autism Spectrum Disorder Screening of Infants** | Siddiqui, S; Gunaseelan, L; Shaikh, R; Khan, A; Mankad, D; Hamid, MA | 2021 | Exclusion reason: Wrong study design |
| **Leveraging AI for the diagnosis and treatment of autism spectrum disorder: Current trends and future prospects** | Wankhede, N; Kale, M; Shukla, M; Nathiya, D; Roopashree, R; Kaur, P; Goyanka, B; Rahangdale, S; Taksande, B; Upaganlawar, A; Khalid, M; Chigurupati, S; Umekar, M; Kopalli, SR; Koppula, S | 2024 | Exclusion reason: Wrong study design |
| **Machine learning for autism spectrum disorder diagnosis using structural magnetic resonance imaging: Promising but challenging** | Bahathiq, RA; Banjar, H; Bamaga, AK; Jarraya, SK | 2022 | Exclusion reason: Wrong study design |
| **Brain imaging-based machine learning in autism spectrum disorder: methods and applications** | Xu, M; Calhoun, V; Jiang, RT; Yan, WZ; Sui, J | 2021 | Exclusion reason: Wrong study design |
| **Application and research progress of machine learning in the diagnosis and treatment of neurodevelopmental disorders in children** | Song, C; Jiang, ZQ; Liu, D; Wu, LL | 2022 | Exclusion reason: Wrong study design |
| **Advances in Autism Spectrum Disorder (ASD) Diagnostics: From Theoretical Frameworks to AI-Driven Innovations** | Syriopoulou-Delli, CK | 2025 | Exclusion reason: Wrong study design |
| **The differential diagnosis of autism spectrum disorder in adults** | Carroll, HM; Thom, RP; McDougle, CJ | 2025 | Exclusion reason: Wrong study design |
| **Unraveling Down Syndrome: From Genetic Anomaly to Artificial Intelligence-Enhanced Diagnosis** | Koul, AM; Ahmad, F; Bhat, A; Aein, QU; Ahmad, A; Reshi, AA; Kaul, RUR | 2023 | Exclusion reason: Wrong study design |
| **Artificial Intelligence: the "Trait D'Union" in Different Analysis Approaches of Autism Spectrum Disorder Studies** | Marciano, F; Venutolo, G; Ingenito, CM; Verbeni, A; Terracciano, C; Plunk, E; Garaci, F; Cavallo, A; Fasano, A | 2021 | Exclusion reason: Wrong study design |
| **Utilising Artificial Intelligence (AI) in the Diagnosis of Psychiatric Disorders: A Narrative Review** | Khare, M; Acharya, S; Shukla, S; Harshita; Sachdev, A | 2024 | Exclusion reason: Wrong study design |
| **Identification of autism spectrum disorder using electroencephalography and machine learning: a review** | Ranaut, A; Khandnor, P; Chand, T | 2024 | Exclusion reason: Wrong study design |
| **Can Autism Be Diagnosed with Artificial Intelligence? A Narrative Review** | Chaddad, A; Li, JL; Lu, QZ; Li, YJ; Okuwobi, IP; Tanougast, C; Desrosiers, C; Niazi, T | 2021 | Exclusion reason: Wrong study design |
| **Artificial intelligence in positive mental health: a narrative review** | Thakkar, A; Gupta, A; De Sousa, A | 2024 | Exclusion reason: Wrong study design |
| **New Strategies for Clinical Trials in Autism Spectrum Disorder** | Pauly, R; Ziats, CA; Abenavoli, L; Schwartz, CE; Boccuto, L | 2021 | Exclusion reason: Wrong study design |
| **Harnessing the potential of human induced pluripotent stem cells, functional assays and machine learning for neurodevelopmental disorders** | Yang, ZQ; Teaney, NA; Buttermore, ED; Sahin, M; Afshar-Saber, W | 2025 | Exclusion reason: Wrong study design |
| **Bringing machine learning to research on intellectual and developmental disabilities: taking inspiration from neurological diseases** | Gupta, C; Chandrashekar, P; Jin, T; He, CF; Khullar, S; Chang, Q; Wang, DF | 2022 | Exclusion reason: Wrong study design |
| **A snapshot on introspection of autism spectrum disorder** | Kale, G; Addepalli, V; Joshi, S | 2024 | Exclusion reason: Wrong study design |
| **Early Diagnosis of Autism Spectrum Disorder: A Review and Analysis of the Risks and Benefits** | Okoye, C; Obialo-Ibeawuchi, CM; Obajeun, OA; Sarwar, S; Tawfik, C; Waleed, MS; Wasim, AU; Mohamoud, I; Afolayan, AY; Mbaezue, RN | 2023 | Exclusion reason: Wrong study design |
| **AI-enabled clinical decision support tools for mental healthcare: A product review** | Kleine, AK; Kokje, E; Hummelsberger, P; Lermer, E; Schaffernak, I; Gaube, S | 2025 | Exclusion reason: Wrong study design |
| **AI-Enhanced Dyscalculia Screening: A Survey of Methods and Applications for Children** | Bhushan, S; Arunkumar, S; Eisa, TAE; Nasser, M; Singh, AK; Kumar, P | 2024 | Exclusion reason: Wrong study design |
| **Diagnosis of mental disorders using machine learning: Literature review and bibliometric mapping from 2012 to 2023** | Sharma, CM; Chariar, VM | 2024 | Exclusion reason: Wrong study design |
| **Video-Based Automatic Baby Motion Analysis for Early Neurological Disorder Diagnosis: State of the Art and Future Directions** | Leo, M; Bernava, GM; Carcagnì, P; Distante, C | 2022 | Exclusion reason: Wrong study design |
| **Early Identification of Language Disorders Using Natural Language Processing and Machine Learning: Challenges and Emerging Approaches** | Lammert, JM; Roberts, AC; Mcrae, K; Batterink, LJ; Butler, BE | 2025 | Exclusion reason: Wrong study design |
| **Autism Data Classification Using AI Algorithms with Rules: Focused Review** | Alsbakhi, A; Thabtah, F; Lu, J | 2025 | Exclusion reason: Wrong study design |
| **Machine Learning Techniques for the Diagnosis of Attention-Deficit/Hyperactivity Disorder from Magnetic Resonance Imaging: A Concise Review** | Periyasamy, R; Vibashan, VS; Varghese, GT; Aleem, MA | 2021 | Exclusion reason: Wrong study design |
| **Review of Progress in Diagnostic Studies of Autism Spectrum Disorder Using Neuroimaging** | Kaur, P; Kaur, A | 2023 | Exclusion reason: Wrong study design |
| **Epigenomic signatures reveal mechanistic clues and predictive markers for autism spectrum disorder** | LaSalle, JM | 2023 | Exclusion reason: Wrong study design |
| **Can Deep Learning Hit a Moving Target? A Scoping Review of Its Role to Study Neurological Disorders in Children** | Sargolzaei, S | 2021 | Exclusion reason: Wrong study design |
| **Unveiling the Diagnostic Potential of Linguistic Markers in Identifying Individuals with Parkinson's Disease through Artificial Intelligence: A Systematic Review** | Palmirotta, C; Aresta, S; Battista, P; Tagliente, S; Lagravinese, G; Mongelli, D; Gelao, C; Fiore, P; Castiglioni, I; Minafra, B; Salvatore, C | 2024 | Exclusion reason: Wrong patient population |
| **Neuroimaging genetics approaches to identify new biomarkers for the early diagnosis of autism spectrum disorder** | Nisar, S; Haris, M | 2023 | Exclusion reason: Wrong study design |
| **A review of methods for classification and recognition of ASD using fMRI data** | Feng, WB; Liu, GY; Zeng, KL; Zeng, MC; Liu, Y | 2022 | Exclusion reason: Wrong study design |
| **Channels and Features Identification: A Review and a Machine-Learning Based Model With Large Scale Feature Extraction for Emotions and ASD Classification** | Aslam, AR; Hafeez, N; Heidari, H; Altaf, MAB | 2022 | Exclusion reason: Wrong study design |
| **Clinical research on neurological and psychiatric diagnosis and monitoring using wearable devices: A literature review** | Huang, JL; Wang, HD; Wu, QH; Yin, J; Zhou, HW; He, Y | 2024 | Exclusion reason: Wrong study design |
| **Resolving heterogeneity in transcranial electrical stimulation efficacy for attention deficit hyperactivity disorder** | Lipka, R; Ahlers, E; Reed, TL; Karstens, MI; Nguyen, V; Bajbouj, M; Kadosh, RC | 2021 | Exclusion reason: Wrong study design |
| **Evidence based Medicine Biomarkers of Neuroelectrophysiology for Mental Disorders** | Tang, XR; Wang, XG; Chen, TL; Zhang, MC; Zheng, Z; Luo, YJ; Gong, QY | 2021 | Exclusion reason: Wrong study design |
| **Artificial intelligence for precision medicine in neurodevelopmental disorders** | Uddin, M; Wang, YJ; Woodbury-Smith, M | 2019 | Exclusion reason: Wrong study design |
| **Role of deep learning in infant brain MRI analysis** | Mostapha, M; Styner, M | 2019 | Exclusion reason: Wrong study design |
| **Advancing rare neurological disorder diagnosis: Addressing challenges with systematic reviews and AI-driven MRI meta-trans learning framework for neurodegenerative disorders** | Gupta, A; Malhotra, D | 2025 | Exclusion reason: Wrong patient population |
| **Age of machine learning: new trends in autism spectrum disorder prediction** | Xu, WH; Li, HB; Li, JW; Jin, M | 2025 | Exclusion reason: Wrong study design |
| **Explainable AI in early autism detection: a literature review of interpretable machine learning approaches** | Agrawal, R | 2025 | Exclusion reason: Wrong study design |
| **Applications of Supervised Machine Learning in Autism Spectrum Disorder Research: a Review** | Hyde, KK; Novack, MN; LaHaye, N; Parlett-Pelleriti, C; Anden, R; Dixon, DR; Linstead, E | 2019 | Exclusion reason: Wrong study design |
| **Machine learning (ML) for the diagnosis of autism spectrum disorder (ASD) using brain imaging** | Nogay, HS; Adeli, H | 2020 | Exclusion reason: Wrong study design |
| **Machine learning in autistic spectrum disorder behavioral research: A review and ways forward** | Thabtah, F | 2019 | Exclusion reason: Wrong study design |
| **Early Autism Screening: A Comprehensive Review** | Thabtah, F; Peebles, D | 2019 | Exclusion reason: Wrong study design |
| **Monitoring cognition in people with epilepsy and intellectual disability** | Catalán-Aguilar, J; Witt, JA; Helmstaedter, C | 2025 | Exclusion reason: Wrong study design |
